# Leaf nodule endosymbiotic *Burkholderia* confer targeted allelopathy to their *Psychotria* hosts

**DOI:** 10.1038/s41598-021-01867-2

**Published:** 2021-11-17

**Authors:** Antri Georgiou, Simon Sieber, Chien-Chi Hsiao, Tatyana Grayfer, Jacob L. Gorenflos López, Karl Gademann, Leo Eberl, Aurélien Bailly

**Affiliations:** 1 Institute of Plant and Microbial Biology, University of Zürich, Zollikerstrasse 107, 8008 Zürich, Switzerland; 2 Department of Chemistry, University of Zürich, Winterthurerstrasse 190, 8057 Zurich, Switzerland

**Keywords:** Cell wall, Plant cell biology, Plant symbiosis, Plant development, Plant morphogenesis, Root apical meristem, Symbiosis

## Abstract

After a century of investigations, the function of the obligate betaproteobacterial endosymbionts accommodated in leaf nodules of tropical *Rubiaceae* remained enigmatic. We report that the α-d-glucose analogue (+)-streptol, systemically supplied by mature *Ca.* *Burkholderia kirkii* nodules to their *Psychotria* hosts, exhibits potent and selective root growth inhibiting activity. We provide compelling evidence that (+)-streptol specifically affects meristematic root cells transitioning to anisotropic elongation by disrupting cell wall organization in a mechanism of action that is distinct from canonical cellulose biosynthesis inhibitors. We observed no inhibitory or cytotoxic effects on organisms other than seed plants, further suggesting (+)-streptol as a *bona fide* allelochemical. We propose that the suppression of growth of plant competitors is a major driver of the formation and maintenance of the *Psychotria–Burkholderia* association. In addition to potential agricultural applications as a herbicidal agent, (+)-streptol might also prove useful to dissect plant cell and organ growth processes.

## Introduction

The leaves of discrete genera of *Rubiaceae* and *Primulaceae* host *Burkholderia* symbionts in their mesophyll and form stoma-derived structures, termed nodules, in a limited number of species^1–3^. Despite the early description of these associations^4,5^, the bacterial contribution to the plant host remains obscure. Leaf-nodulating *Psychotria* are restricted to *ca.* 80 monophyletic tropical African species and have been thoroughly investigated^1,6,7^. A pool of host-specific endosymbionts is maintained into shoot meristems; this bacterial population colonizes reproductive organs and leaves as they emerge. Mature flowers (hence seeds) inherit a small *Burkholderia* population that perpetuates the symbiotic cycle; a unique case of vertical transmission in higher plants^1,8^. The incongruent phylogenies of the *Burkholderia* symbionts and their *Rubiaceae* hosts advocate for occasional, presumably insect-driven, horizontal transmission; however, extant partners do not survive outside of the symbiosis^9,10^. *Burkholderia* cells proved unviable in axenic cultures and aposymbiotic plants die within months due to the collapse of the shoot apical meristems. The transition from a free-living lifestyle to an obligate symbiont was accompanied by a massive erosion of the *Burkholderia* genomes^1,11,12^. Genomic and proteomic evidence ruled out historical speculations about the role of the *Psychotria* endosymbionts, including nitrogen fixation or phytohormones production^11,12^. Yet, neither the molecular mechanisms triggering and maintaining the symbiosis nor the reason of the obligate nature of the symbiosis have been elucidated.

Interestingly, in the context of genetic drift, the genome of *Candidatus* *Burkholderia kirkii* has retained a 140-kb low copy number plasmid (pKIR01)^11^, which is absent from free-living *Burkholderiaceae.* However, pKIR01 orthologous genes are also found in other *Psychotria* leaf nodule symbionts^10^. Their conservation underlines their importance for the symbiosis. The plasmid pKIR01 encodes enzymes with significant homology to genes involved in the biosynthesis of C_7_ cyclitol-containing secondary metabolites in Actinobacteria^11^. Bioactivities of such compounds include glycosidase inhibition (acarbose), and antitumoral (pericosine A), antifungal (validamycin A), and insecticidal (validoxylamine A) properties^13,14^.

In contradiction to former reports suggesting that *Burkholderia* cells shift to a dormant state in mature nodules^15^, the pKIR01 2-epi-5-epi-valiolone synthase (EEVS), catalysing the committed step in cyclitols biosynthesis, was shown to be highly induced in nodules relative to the apical population^12^. Furthermore, the high abundance of pKIR01 proteins assigned to cyclitols production suggests that *Candidatus* *B. kirkii* dedicates its metabolism to the production of bioactive compounds once nodules are established. Indeed, two C_7_ cyclitols found in high amounts in the nodulated leaves of *Psychotria kirkii*, but absent from aposymbiotic plants, were recently isolated and synthesized^10,16,17^. The *N*-acetyl glucosamine analogue kirkamide is toxic to arthropods, thus hinting towards a protective role against folivorous insects^16^. In addition, kirkamide and related compounds inhibit multiple *N*-acetyl-d-glucosaminidases^18^. The pseudodisaccharide (+)-streptol-β-glucoside (SG) and its aglycone moiety (+)-streptol were shown to inhibit lettuce germination^10,17,19^. The exclusive distribution and conservation of the cyclitol biosynthetic genes in leaf-nodule *Burkholderia*, supported by the presence of the compounds in host plants^10^, argues for positive selection of these traits and suggests a defensive symbiosis.

Separating chemical interference from other mechanisms, such as resources or spatial competition, has proven difficult and the role of secondary metabolites-based allelopathy in evolutionary ecology is currently under debate^20^. In this study, we investigate the contribution of C_7_ cyclitols to the *P. kirkii* chemical arsenal against plant competitors. This is the first report of a natural antagonist that targets root growth of seed plants.

## Results

### Allelopathic activity of *P. kirkii* leaf extracts

We used the high sensitivity of lettuce seedlings towards symbiotic *P. kirkii* leaf extracts^10^ as a benchmark for evaluating the inhibitory activity within distinct plant organs. Lettuce germination was greatly hindered by filtered water extracts of *P. kirkii* apical shoots, flowers, fruits, hypocotyls and roots (Supplementary Fig. 1a). In addition, lettuce seeds did not grow in the presence of intact mature drupes but developed when mesocarps were removed from pyrenes (Supplementary Fig. 1b). Previous work has quantified high concentration of kirkamide and SG, but low concentration of streptol in *P. kirkii* leaves, shoots and roots^10,17^. Thus the cyclitols appear systemically distributed throughout *P. kirkii*. Allelochemicals can be released into the environment as volatiles, root exudates, leachates of aboveground parts or by decomposition of plant material. We detected streptol (0.14 ± 0.001 µg ml^−1^) in the root exudates of axenically grown symbiotic *P. kirkii*, but not of aposymbiotic plants. Leachates obtained by bathing intact *P. kirkii* leaves in water contained sufficient inhibitory activity to prevent lettuce root establishment, but did not completely block germination, as embryo roots protruded from the seed coat and hypocotyls developed similarly in treated and untreated seedlings (Supplementary Fig. 1c). In fact, lettuce samples germination displayed a binary response towards *P. kirkii* leaf extracts, suggesting that inhibition of root development requires a threshold concentration of the inhibitor. We further evaluated the allelopathic potential by growing black mustard seeds on soil watered with *P. kirkii* leaf leachates or crude extract dilutions. Surprisingly, the seedlings germinated and developed intact aerial organs when the soil was kept moist; yet, root length was drastically affected in both treatments (Supplementary Fig. 2a,c). Closer inspection of these roots revealed swollen tissues above the root apical meristem (Supplementary Fig. 2b). In vitro mustard germination rates were not affected by the leachate but the success of seedlings to establish in soil greatly dropped with lower water supply (Supplementary Fig. 2d). Similar results were obtained with mustard seeds sown on soil amended with mulched *P. kirkii* leaves. These combined results indicated that symbiotic *P. kirkii* plants contain and release a potent allelochemical that inhibits root development.

### *Psychotria kirkii *extract specifically impedes root elongation

**Figure 1:**
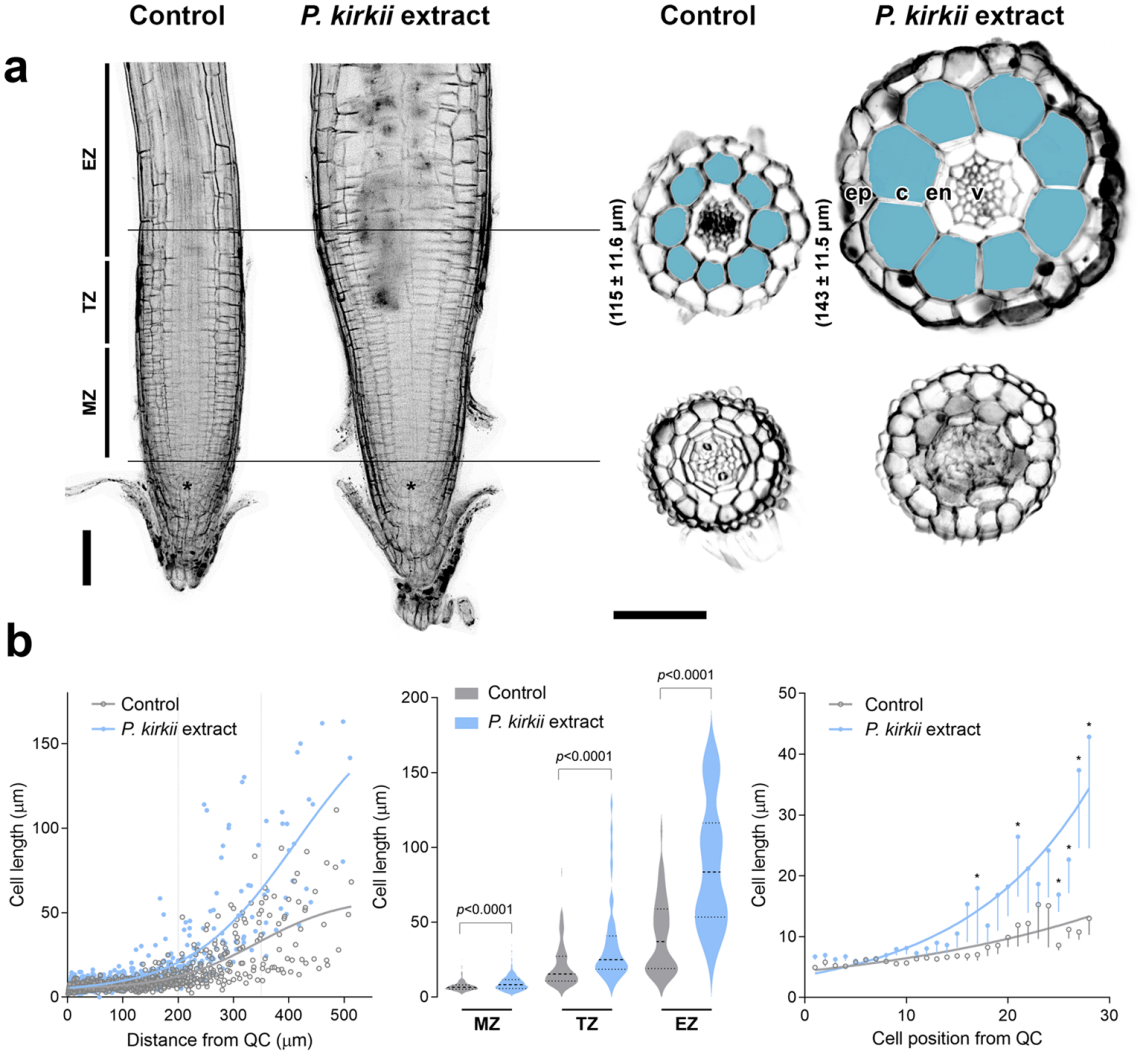
*P. kirkii* extracts disrupt root anisotropic growth. (**a**) Left, optical longitudinal sections of mPS-PI-stained Arabidopsis roots. Progressive swelling of treated root cells transitioning to the elongation zone is apparent. Asterisks mark the root quiescent centre. Bar = 100 µm. Right, corresponding cross sections highlighting the epidermal and cortical radial expansion in treatment. Numbers in brackets indicate the mean of section diameters with standard deviation (n = 12). *v* vasculature, *en* endodermis, *c* cortex, *ep* epidermis. Bar = 100 µm. (**b**) Left, cortical cell length in function of the distance to the quiescent center (QC). Dataset represents fifteen seedlings per treatment, curves were fitted with the Gompertz model, circles indicate data distribution. Dotted lines delimit the distinct apical root zones. Middle, cortical cell length in the given root apical zone. Significant differences between treatment and water control were analysed in two-sided unpaired t-tests (*p*-values are indicated). Right, cortical cell length in function of the position from QC initials. Data represent mean values; curves were fitted with the exponential growth model, standard errors are indicated. Significant differences between treatment and untreated control for each position were analysed in two-sided unpaired t-tests (*p-value < 0.05). *MZ* meristematic zone, *TZ* transition zone, *EZ* elongation zone.

To clarify the biological activity of *P. kirkii* extracts, we took advantage of the well-studied model plant *Arabidopsis thaliana*. In vitro, *Arabidopsis* seedlings germinated and developed to maturity in the presence of up to 15 ppm *P. kirkii* extracts (Supplementary Fig. 3a). Aerial organs did not show defects or changes in stature when compared to controls (Supplementary Fig. 3b); however, the root system appeared extremely branched and stunted. Doubling the concentration of *P. kirkii* extracts in the medium was sufficient that *Arabidopsis* seedlings formed dwarves (Supplementary Fig. 3a). Time-lapse monitoring of *Arabidopsis* germination and growth on vertical plates supplemented with *P. kirkii* extracts confirmed that the active compounds rapidly inhibited root elongation, but did not impede seed germination or lateral root emergence (Supplementary Fig. 4a,b). We noticed that this inhibition was diminished when 1% sucrose was added to the growth medium (Supplementary Fig. 4d). Transferring control-grown seedlings to medium supplemented with 15 ppm *P. kirkii* extracts quickly stalled root growth (Supplementary Fig. 4c), confirming the organ as the prime target. Thus, the active compounds act as pre-emergence root elongation inhibitors. Microscopic inspection of treated roots revealed a radial swelling of the epidermal and cortical tissues above the meristematic zone (Fig. 1a). Measurements of longitudinal root cell lengths from the quiescent centre in the cortical files showed that cells treated with *P. kirkii* extracts transitioned earlier from the meristematic (MZ) to the elongation zone (EZ) compared to controls (Fig. 1b). The 10–15 most distal meristematic cells showed equivalent dimensions in controls and treatment, however treated cells entering the transition zone (TZ) quickly elongated while enlarging. By contrast, treated cells protruding the EZ rarely grew longer than 100 µm at maturity. To dismiss that the swelling was due to an increased number of actively dividing cells, we monitored the expression pattern of the cell cycle marker pCYCB1;1::DB‐GUS^21^ in seedlings transferred to *P. kirkii* extracts. Symptomatic roots exhibited similar numbers of GUS-positive cells and lengths of pCYCB1;1::DB‐GUS expression regions as control roots; no ectopic cell divisions were detected (Supplementary Fig. 5a). In order to assess if the observed cell enlargement is caused by incomplete cytokinesis, we carefully monitor primary cell wall integrity and nuclear partitioning along the root apex zones (Supplementary Fig. 5b,c). Symptomatic seedlings did not present abnormal ploidy or incomplete cell plates in the affected EZ or in the MZ, suggesting that cytokinesis occurred normally despite isotropic growth. Also, we did not observe differences in the levels or the distribution of auxin in treated root tips versus control seedlings (Supplementary Fig. 5d). Taken together, these data indicate that *P. kirkii* extracts do not primarily impact cell proliferation nor root patterning but appears to disturb root growth anisotropy at the onset of cellular elongation.

### The α-d-glucose analogue (+)-streptol is the active pharmacophore

**Figure 2:**
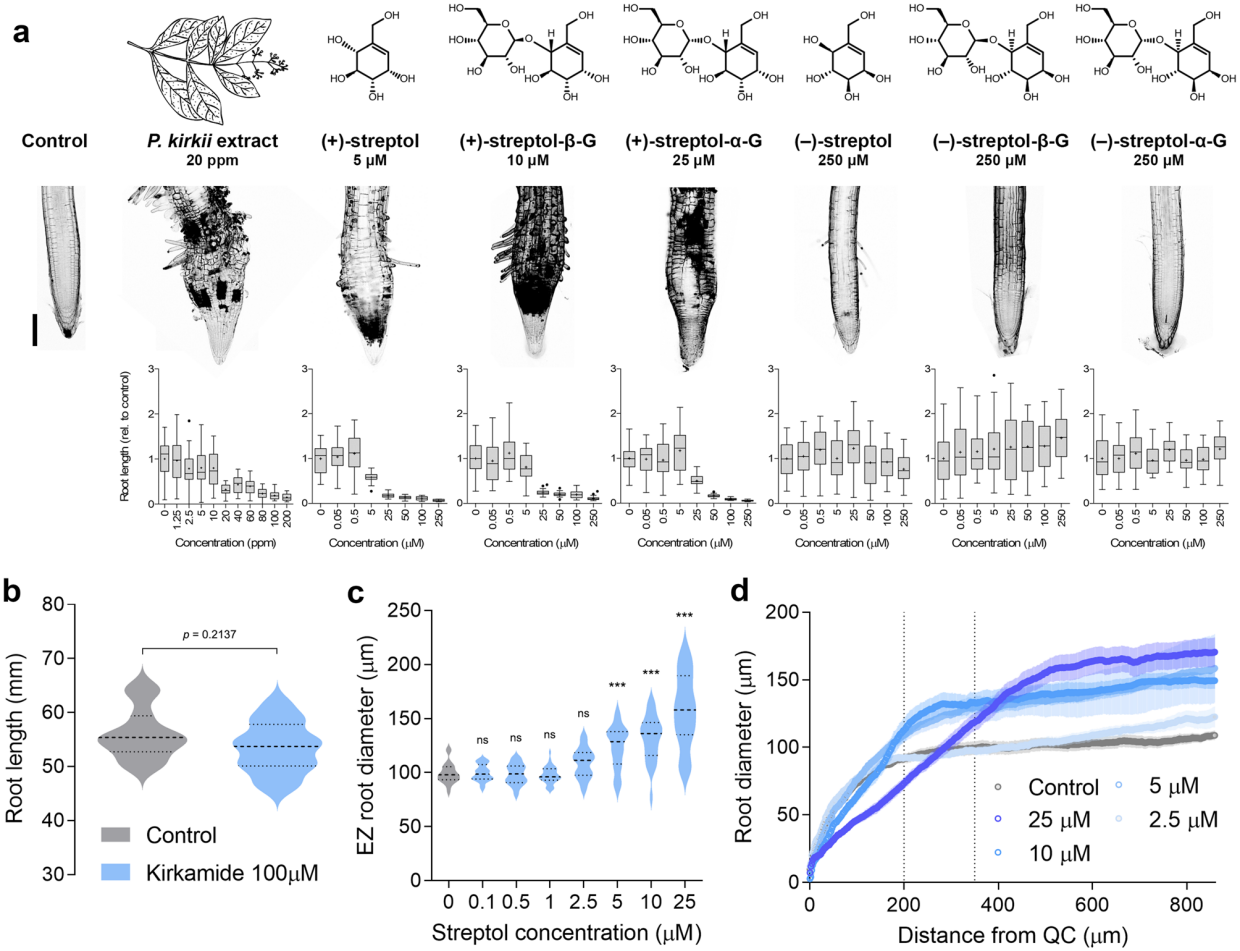
Streptol defines the *P. kirkii* active pharmacophore. (**a**) Arabidopsis root growth inhibition by synthetic, conjugated and enantiomeric streptol derivatives. Root micrographs illustrate the apical phenotype at the IC_50_ or highest tested dose. Bar = 100 µm. Boxplots represent root length relative to water controls. + indicates the mean values of three biological replicates (10 seedlings each); dots indicate outliers. (**b**) Root length of 10 days old Arabidopsis seedlings grown in the presence 100 µM kirkamide or without supplementation. Significant differences between treatment and water control were analysed in a two-sided unpaired t-test. Values represent 12 seedlings, *p*-value is indicated. (**c**) Arabidopsis root diameter in the elongation zone (EZ) after 5 days of growth in increasing concentrations of streptol. Significant differences between treatment and water control were analysed in a one-way ANOVA with Dunnett’s post-hoc test (***p-value < 0.001; ns, not significant). Data represent the values of three biological replicates (10 seedlings each). (**d**) Root apex diameter along the longitudinal axis of 5 days old seedlings grown in increasing concentrations of streptol. Data represent the mean values of 12 seedlings per treatment; standard errors are indicated. Dotted lines delimit the distinct apical root zones. *QC* apical quiescent centre.

We next tested the bioactivity of pure naturally-produced as well as synthetic derivatives of cyclitols^17^ (Fig. 2a). The natural products (+)-streptol, (+)-streptol-β-glucoside and (+)-streptol-α-glucoside (A-79197-2^22^) inhibited root development with apparent IC_50_ values of 4.7 ± 1.1 s.e., 9.4 ± 1.1 s.e. and 16.7 ± 1.2 s.e. µM, respectively. Importantly, (−)-streptol, (−)-streptol-β-glucoside and (−)-streptol-α-glucoside (diastereomer of A-79197-2) did not exhibit bioactivity, suggesting that the active compounds may target a specific protein. High concentration of kirkamide (100 µM) had also no effect (Fig. 2b). The dose-dependent radial expansion of root tips upon (+)-streptol treatments confirmed that 5 µM was sufficient to mimic the effects of *P. kirkii* extracts (Fig. 2c,d). Beside a two-fold change in activity, both (+)-streptol and SG showed comparable effects on *Arabidopsis* photo- and skotomorphogenic development and treatments with these compounds led to root growth dose–response curves with steep slopes (Supplementary Fig. 6a). Etiolated *Arabidopsis* hypocotyls did not show abnormalities in the rapidly-elongating apical hook region, suggesting that (+)-streptol targets cells entering into the root EZ.

We previously hypothesized that streptol glycosylation might lower the toxicity of the compound for the host plant^17^. As checkerboard assays did not reveal synergism between (+)-streptol and SG (Supplementary Fig. 6b), we speculated that SG could act as a prodrug. Similar amounts of (+)-streptol and SG were detected in the roots of *Arabidopsis* mature plants grown on 15 ppm *P. kirkii* extracts (0.44 ± 0.01 and 0.53 ± 0.02 µg ml^−1^ extracts, respectively); however, only (+)-streptol (0.31 ± 0.01 µg ml^−1^) was detected in inflorescences. To support these results, we bathed mature *Arabidopsis* roots in a solution of 250 µM pure SG for 48 h. After careful washing, we detected substantial amounts of (+)-streptol (0.85 ± 0.06 µg ml^−1^) in these roots, but not of SG. Similarly, we found that the etiolated hypocotyls of *Arabidopsis* grown on medium supplemented with 250 µM SG accumulated 10 times more (+)-streptol than SG (3.98 ± 0.03 and 0.44 ± 0.01 µg ml^−1^, respectively). Interestingly, when control-grown mature *Arabidopsis* roots were transferred to solid medium supplemented with 100 µM SG, a significant amount of (+)-streptol was found in the gelose medium after 10 days (0.76 ± 0.03 µg ml^−1^). (+)-Streptol was not detected in the medium in the absence of roots.

**Figure 3:**
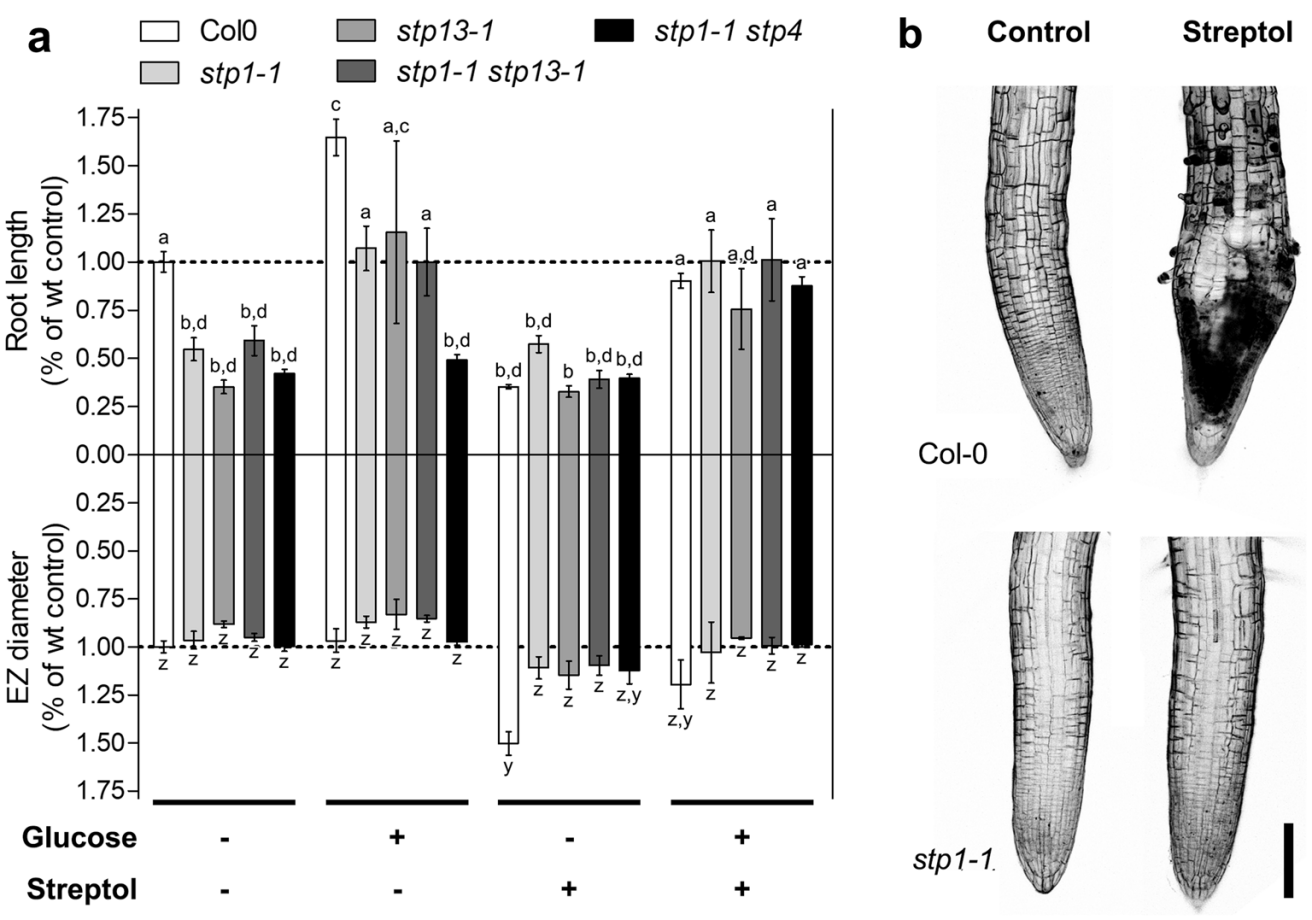
Streptol is transported by STP hexose symporters. (**a**) Root length and elongation zone diameter of wildtype and mutant seedlings grown in the presence or absence of 5 µM streptol and 2.5 mM d-glucose; values are relative to wildtype water controls. Significant differences between samples were analysed in a one-way ANOVA with Tukey’s post-hoc test; significantly different means (*p*-value < 0.05) are marked with different letters. Data represent the mean values of at least three biological replicates; standard errors are indicated. (**b**) Representative micrographs of wildtype and *stp1* mutants root apex phenotype after 5 days of growth in 5 µM streptol. Bar = 100 µm.

Collectively, these data suggest that (+)-streptol and/or SG can be taken up by the roots and that at least (+)-streptol is systemically transported *in planta*. It is worth noting that the lack of toxicity on aerial organs is not due to the absence of (+)-streptol in these tissues. Given (+)-streptol structural proximity to α-d-glucose, the translocation might occur through dedicated plasmalemma transporters. In *Arabidopsis*, the major facilitator superfamily SUGAR TRANSPORT PROTEIN (STP) symporters mediate the uptake of various hexoses^23,24^. We found single and double mutants in the root expressed STP1, 4 and 13 insensitive to restrictive doses of (+)-streptol (Fig. 3). Exogenous supplementation of glucose restored root growth in STP-defective lines and substantially alleviated streptol symptoms (Fig. 3a). Similar results were obtained using 10 µM SG. We concluded that (+)-streptol primarily enters and translocates into the plant body via hexose transporters.

### Streptol specifically disturbs root cell wall organisation without affecting cellulose biosynthesis

Several carbohydrate-related genetic lesions in *Arabidopsis* cause extensive swelling of the root apex. In particular, mutations and pharmacological interference obstructing cell wall biosynthesis and remodelling trigger phenotypes resembling those observed in (+)-streptol conditions^25–28^.

The primary plant cell wall is a polysaccharidic extracellular matrix synthesized and rearranged to support cell morphogenesis and growth under vacuole-generated turgor pressure^29^. The major carbohydrate polymers of primary walls are cellulose, hemicelluloses and pectins^30^. Cellulose, a linear β‐(1,4)‐linked glucan chain, is synthesized at the plasma membrane by multi-heteromeric complexes termed Cellulose Synthase Complexes (CSCs)^31^. Primary wall CSCs are composed of at least three distinct CELLULOSE SYNTHASE (CesA) isoforms^32,33^, CESA1 and CESA3 operating as core units, and context-specific CESA6‐like proteins complementing the structure^34–36^. CSCs are superorganized in hexameric structures known as “rosettes”^31,37^ that are guided along cortical microtubules to achieve directional cellulose deposition into the apoplast^38–40^. The multiple cellulose chains produced by CSCs arrange into higher order, H bond-driven microfibrils perpendicular to the growth axis, thus defining anisotropic elongation^41,42^. These microfibrils are tethered by hemicelluloses and embedded into a pectic matrix that confers optimal viscoelasticity to the cell wall. These polymers are produced in the Golgi apparatus and released to the apoplast by exocytosis.

**Figure 4:**
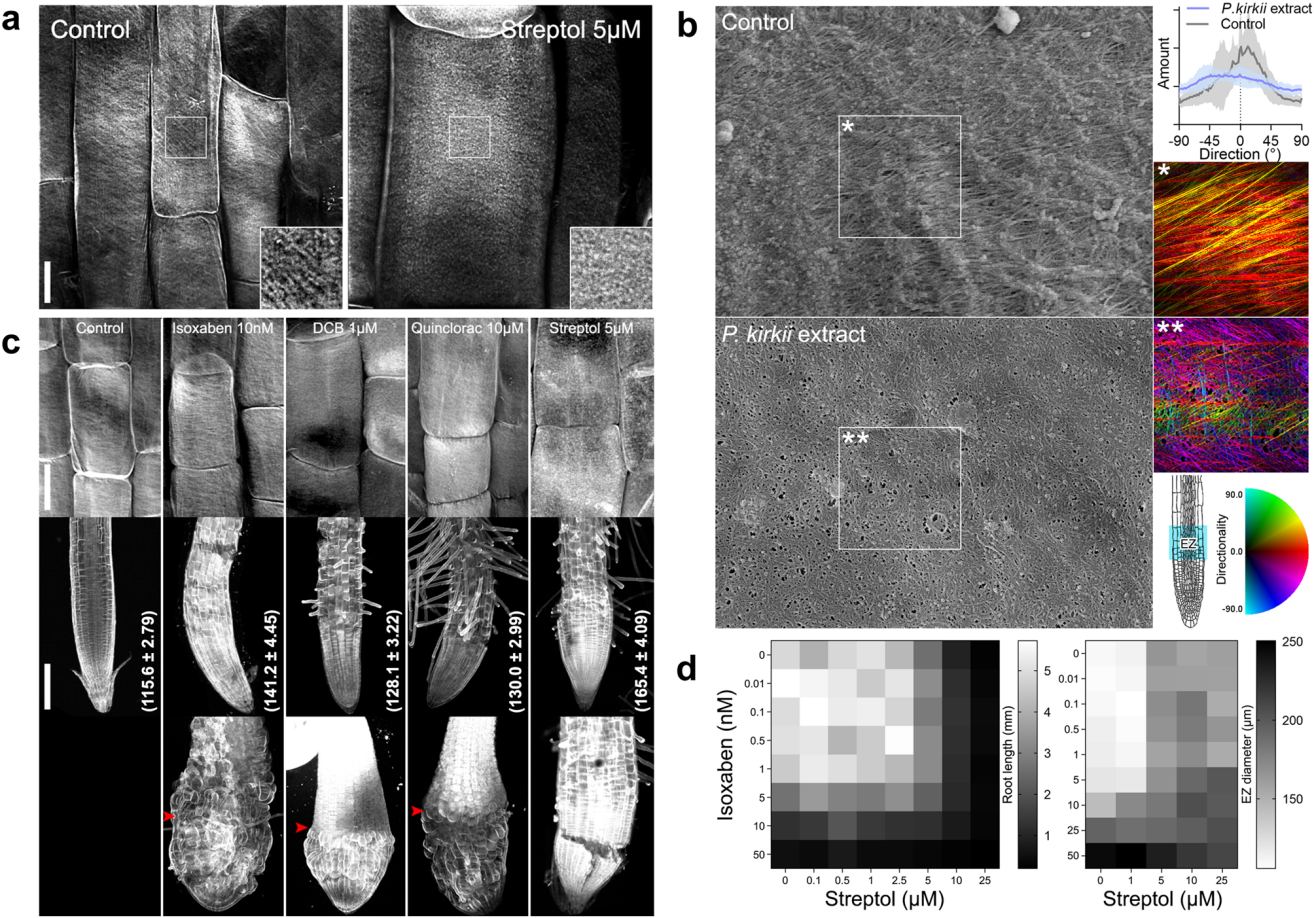
Streptol disorganizes plant cell walls in elongating root cells without inhibiting cellulose production. (**a**) Direct Red 23 cellulose staining of rapidly elongating epidermal EZ cells in presence or absence of 5 µM streptol. Insets show twofold magnification of the signal. Maximum projections of CLSM optical sections. Note that the same scale and acquisition settings were used for both samples. Bar = 10 µm. (**b**) Scanning electron micrographs of the EZ external epidermal surface of control and *P. kirkii* extract-treated roots; 50kX magnification. Insets marked with asterisks depict the local Fourier components orientation map, color-coded as in the HSB wheel according to angle, power spectrum and image grey values, respectively. Top right, directionality histograms distribution of cellulose microfibrils. Data represent the mean values of three biological replicates (five cell surface samplings per seedling), standard deviations are indicated. (**c**) Direct Red 23 cellulose staining of root apices in presence or absence of the given inhibitors. Top, first elongating EZ cells. Bar = 10 µm. Middle, corresponding root apex phenotypes. Numbers in brackets indicate mean EZ diameter and standard errors in micrometres (15 seedlings each). The means of each treatment were found significantly different from water control (one-way ANOVA with Dunnett’s post-hoc test, *p*-value < 0.05). Bar = 100 µm. Bottom, root apex phenotypes at root growth-restrictive concentrations. Arrowheads indicate the root-shoot junction. Maximum projections of CLSM optical sections. The same acquisition settings were used for all samples. Bar = 100 µm. All specimens were oriented to the longitudinal axis. (**d**) Streptol *vs* isoxaben checkerboard root growth and EZ diameter assay. Data represent the mean values of three independent experiments.

In order to understand the mechanism of (+)-streptol-mediated growth inhibition, we assessed the composition of (+)-streptol-treated apical cell walls through an array of histological stains. We first observed higher degrees of dyeing for all histochemicals, suggesting either greater wall polymers contents or better accessibility for the dyes (Supplementary Fig. 7). Under (+)-streptol conditions, Wiesner’s reaction, ruthenium red and basic fuchsin colorations indicated increased and ectopic lignin contents in the EZ, a common trait of cellulose-deficient mutants^43^. Next, outer epidermal cellulose microfibrils arrays in the EZ were observed after Direct Red 23 staining^41^ under the confocal microscope (Fig. 4). Although (+)-streptol treatments did not radically affect the apparent abundance of microfibrils, the characteristic microfibrils transverse orientation of anisotropically growing cells was disordered, which may explain the isotropic expansion of symptomatic cells. The aberrant punctuate distribution of Direct red 23 signals under (+)-streptol treatment (Fig. 4a) strikingly resembles the fluorescence patterns of pectic rhamnogalacturonan-I obtained in root EZ epidermal cells after metabolic labelling with a fucose alkyne analogue^44^. Fourier transformation of scanning electron micrographs of outer epidermal walls in the EZ confirmed that the net directionality of cellulose bundles patterning is heavily altered in the presence of (+)-streptol, with an overall loosening of the matrix lattice (Fig. 4b).

The transversal, CesA-mediated, directional deposition of cellulose microfibrils required for root cell elongation is sustained by cortical microtubules (CMT) alignments^45^. In untreated wild-type roots, CMTs display a predominant transversal orientation throughout the apex^46^. However, the random reorientation of CMTs in the EZ has been consistently reported in cellulose-deficient mutants or upon wall biogenesis chemical perturbation^45^ . Further, this aberrant CMT behaviour occurs in root-swelling mutants and pharmacological CMT interference triggers swollen roots^47,48^. We thus sought to assess the status of epidermal CMTs orientation by monitoring seedlings expressing pUBQ10:EYFP-TUB6 in (+)-streptol conditions. In line with the absence of obvious defects in the MZ, (+)-streptol-treated cells in this zone displayed the canonical transverse CMT orientation (Supplementary Fig. 8a). Contrary to untreated seedlings, cells transitioning to the EZ in the presence of (+)-streptol exhibited a progressive shift of CMT orientations from transverse to random, resulting in a significantly decreased anisotropy (Supplementary Fig. 8a). No differences in CMT behaviour in aerial organs epidermal cells were observed between treatment and control seedlings (Supplementary Fig. 8b).

It is therefore likely that (+)-streptol, directly or indirectly, affects CMT-cell wall organization. However, in contrast to established cellulose biosynthesis inhibitors^49,50^ (CBIs), no experimental evidence supports a direct inhibition of cellulose microfibrils deposition by the cyclitol. The group I isoxaben^51^, group II 2,6-dichlorobenzonitrile^52^ and auxinic quinclorac^53^ CBIs did not cause (+)-streptol-like responses in seedlings (Fig. 4c). At permissive concentrations, these inhibitors produced mild to no relaxation of the EZ epidermal matrix, contrasting with (+)-streptol-triggered defects. Higher restrictive CBIs doses completely impeded root development and often caused additional abnormalities to hypocotyls, symptoms that are absent in (+)-streptol-treated cells (Fig. 4c). Checkerboard assays with increasing doses of (+)-streptol and isoxaben resulted in additive effects in the inhibition of root elongation and EZ swelling (Fig. 4d), suggesting that the two drugs act independently. Moreover, well-investigated CSC mutants such as the CBI-resistant *procuste*^35^ (*prc1*, *cesa6*), *constitutive expression of VSP1*^54^ (*cev1*, *cesa3*), and the anisotropy mutant *radially swollen 1*^55^ (*rsw1*, *cesa1*) were found fully sensitive to (+)-streptol, indicating that the cyclitol targets a different component of the cell wall biosynthesis apparatus (Supplementary Fig. 9a). We also evaluated the crystalline cellulose content in cells from the symptomatic EZ and found no compelling evidence of significant changes between control and treatment (Supplementary Fig. 9b). Furthermore, radial swelling associated with cellulose-defective mutants was reported to be triggered or enhanced by high temperature^26,55^; however, the (+)-streptol phenotype was not enhanced in seedlings transferred to 28 °C (Supplementary Fig. 10). Finally, *Arabidopsis* root cell suspensions cultured for 7 days in presence or absence of 10 µM (+)-streptol did not show significant differences in growth rates or cell shape (Supplementary Fig. 11). Hence, the cyclitol presumably does not directly target the plasma membrane CesAs but interferes with distinct primary cell wall biogenesis or remodelling processes in a cell type- or tissue-specific manner.

### Streptol does not target glycoside hydrolase activity

Glycoside hydrolases (GH) are primarily involved in cell wall degradation^56^ and represent obvious candidate targets of (+)-streptol inhibition. However, several lines of evidence argue against this hypothesis. First, neither (+)-streptol, SG, or their isomers showed inhibition of α- or β-glucosidase activities in vitro (not shown). Blockage of such enzymes, especially starch degrading GHs, would rapidly induce starvation and greater tissue disorganization than observed with (+)-streptol^57,58^. Second, mutations in GHs engaged in N-linked glycan processing such as the *Arabidopsis* alpha-glucosidase II mutant *rsw3*^28^, the embryo defective alpha-glucosidase I mutant *knf-14*^59^ or the α-mannosidase I *mns* multiple mutants^60^, can cause strong reduction in cellulose contents and radial root swellings but exhibit morphological changes that are distinct from streptol treatment. Likewise, micromolar doses of the α-mannosidase I inhibitor kifunensine was shown to trigger acute root swelling in *Arabidopsis* more severe than (+)-streptol effects^60^. By analogy, high micromolar doses of the iminosugars 1-deoxynojirimycin and isofagomine, respectively generic α- and β-glucosidase inhibitors, impede root growth and stimulate radial root expansion but do not alter wall patterning at growth permissive concentrations^57,58^ (Supplementary Fig. 12). Also, the glucose analogue conduritol-β-epoxide (CBE), a covalent inhibitor of animal glucocerebrosidase targeting the *Arabidopsis* glucosylceramidase GCD3^61^, affects root growth without drastically disturbing cell wall organization. Third, *Arabidopsis* single mutants in major cell wall or cytosolic invertases, which are sucrose-degrading GHs, did not respond differently to SG treatments than wild-type plants (Supplementary Fig. 13a). Although no particular cell wall defects were associated with invertase single mutants, the isotropic root growth of a *cinv1 cinv2* double mutant was attributed to an impaired cellulose microfibrils patterning distinct from the one observed under (+)-streptol conditions^62^. Mutant seedlings of the bidirectional sucrose synthase^63^ (SUS1) responded to (+)-streptol and SG similar as the wild-type plant (Supplementary Fig. 13b), in line with the lack of a cell wall phenotype in the SUS quadruple mutant^64^. In conclusion, our data suggest that the two cyclitols do not act as generic GH inhibitors *in planta*.

### Streptol is not a generic antimetabolite and affects only higher plants

Recent work demonstrated the inhibition of 3-dehydroquinate (DHQ) synthase by the cyanobacterial pseudosugar 7-deoxy-sedoheptulofuranose (7dSh)^65^. This antimetabolite blocks the shikimate pathway and thereby triggers growth arrest or cell death in a wide range of prototrophs. *Arabidopsis* development was clearly affected by micromolar doses of 7dSh, although no defects in the root organization were reported^65^. The pentose phosphate pathway intermediate sedoheptulopyranose 7-phosphate is a prominent precursor of cyclitol moieties found in natural products such as acarbose or validamycin A and, interestingly, the EEVS sugar phosphate cyclase is homologous to the DHQ synthase^66^.

In order to evaluate if (+)-streptol features a similar mechanism of action, we first cultured a panel of bacteria in presence of the compounds. Growth kinetics of Gram-negative and Gram-positive organisms were not impacted by 250 µM (+)-streptol in rich or minimal medium (Supplementary Fig. 13a). When grown on solid media, none of the tested bacterial species showed significant changes in colony sizes or extracellular polysaccharides production in the presence of either *P. kirkii* extracts, SG or (+)-streptol (Supplementary Fig. 14a). Finally, the establishment of polysaccharide-rich *Burkholderia* and *Pseudomonas* biofilms was not disturbed by up to 1 mM (+)-streptol (Supplementary Fig. 14b). In our experimental setup, the cyclitols did not demonstrate bactericidal nor bacteriostatic properties.

We next challenged the stramenopile *Phytophthora infestans* with (+)-streptol. The cellulose-based cell wall of *P. infestans* is sensitive to 2,6-dichlorobenzonitrile applications^67^; however, hyphal growth, spore formation and germination were not affected by the presence of *P. kirkii* extracts or micromolar concentrations of (+)-streptol (Supplementary Fig. 15). Similarly, we observed no significant antifungal activity by treatment with *P. kirkii* extracts or with SG and (+)-streptol even at millimolar concentrations (Supplementary Fig. 16). These results argue against streptol-mediated inhibition of a central metabolic pathway in these organisms.

Hatching *Artemia salina* dehydrated eggs in increasing micromolar concentrations of (+)-streptol did not influence cyst decapsulation nor further nauplii growth and development (Supplementary Fig. 17a). (+)-streptol-treated specimens showed no changes in coloration or visible abnormalities. Likewise, we did not observe alterations of human colon carcinoma LS174T cells viability upon (+)-streptol treatment, even at concentrations two orders of magnitude higher than that required for root inhibition (Supplementary Fig. 17b), thus discarding acute cytotoxicity towards animals.

To test whether the inhibitory activity of (+)-streptol is restricted to certain plant lineages, we cultured the single-celled Chlorophyta *Chlamydomonas reinhardtii* and *Gloeotilopsis planctonica* as well as the filamentous Charophyta *Klebsormidium flaccidum* and *Zygnema circumcarinatum* in K-medium supplemented with 10 µM (+)-streptol. After one week of growth, (+)-streptol-treated cultures were indistinguishable from controls. Closer inspection of cell shape and Calcofluor White (CFW) staining of cell walls did not reveal significant alterations in the (+)-streptol-treated populations (Supplementary Fig. 18). We also measured individual cells area from micrographs, as a proxy for cell volume, but did not observe significant increase or decrease in cell sizes for the four tested species (Supplementary Fig. 18). We next extended our survey to mosses. *Physcomitrella patens*, *Funaria hygrometrica* and *Physcomitrium pyriforme* grown for one month on solid medium supplemented with 10 µM (+)-streptol appeared identical to controls in colony size and coloration. No changes were observed in shape and size of organs, including rhizoids, and CFW staining did not reveal alterations in cell wall architecture (Supplementary Fig. 19). Noteworthily, none of the lower plant species tested displayed evident chlorosis, ruling out that streptol blocks central phototrophic pathways.

**Figure 5:**
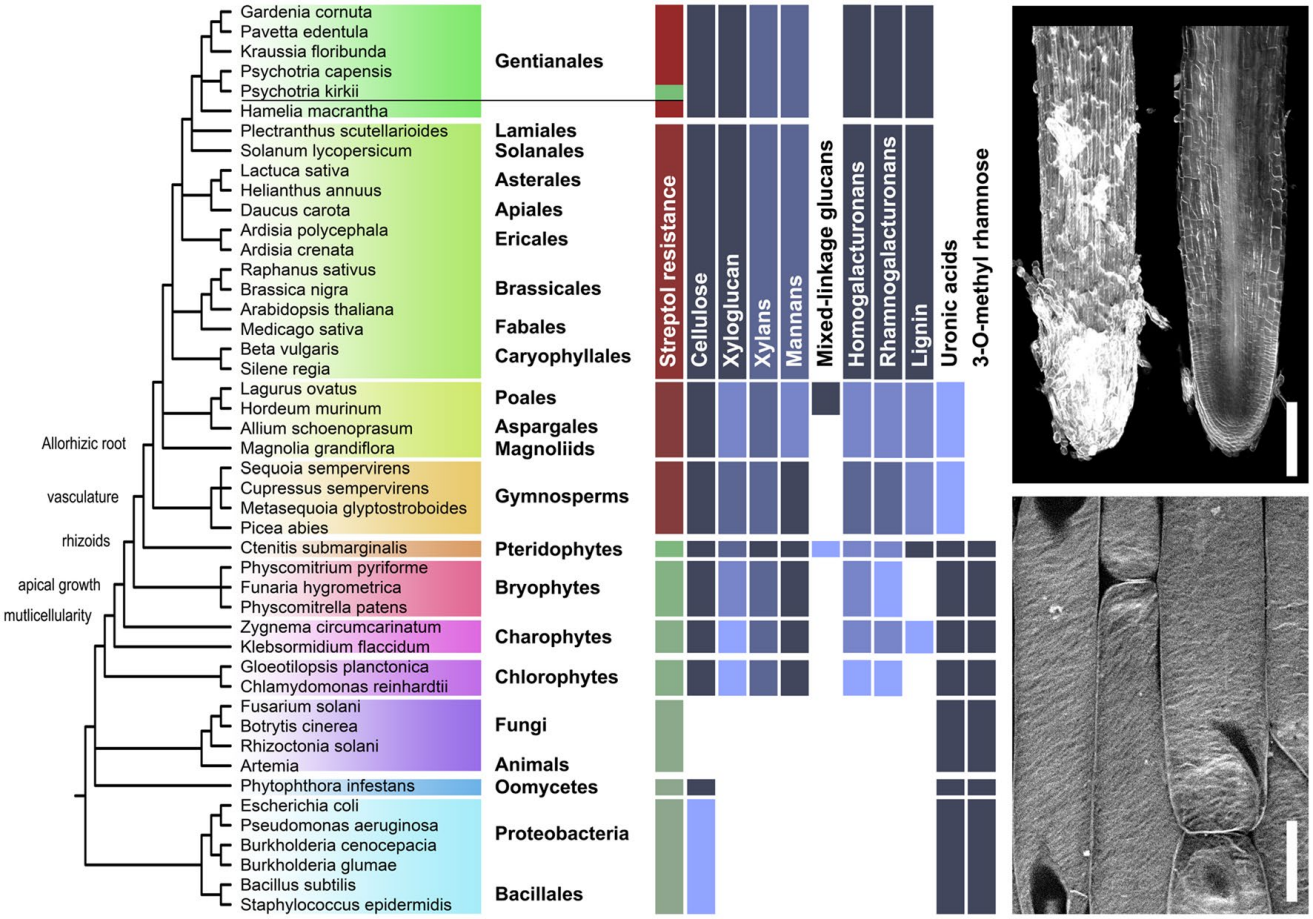
Streptol-mediated growth inhibition is restricted to seed plants. Left, tentative overview of plants cell wall polymers distribution in the organisms tested herein. Shades of blue indicate the relative abundance. Simplified phylogenetic tree based on NCBI taxonomy TAXIDs (not to scale). Species and orders are indicated. Principal root-oriented plant innovations are indicated. Red indicates sensitivity towards *P. kirkii* extracts, green insensitivity. Upper right, external and longitudinal optical section of mPS-PI-stained axenic, nodulated *P. kirkii* fine roots. Note the absence of swelling or root tissue disorder. Bar = 200 µm. Bottom right, Direct Red 23 cellulose staining of *P. kirkii* EZ epidermal cells. Note the canonical, regular alignment of microfibrils. Bar = 20 µm.

To assess if streptol requires components of the complex tissue organization of vascular plants for activity, we germinated mature spores of the Polypodiopsida *Ctenitis submarginalis* on solid medium containing micromolar concentrations of (+)-streptol until the photosynthetic gametophyte stage. No defects in cell division, dimensions or organ patterning were observed and rhizoids grew as much as in untreated samples (Supplementary Fig. 20). However, treatment of seeds from four distinct gymnosperms failed at establishing seedlings when grown in the presence of *P. kirkii* extracts and their roots showed swellings similar to those observed in *Arabidopsis*. To confirm the restriction of streptol effects to spermatophytes, we expanded the germination assays to various angiosperms (Fig. 5). The seeds from all magnoliids, monocots and eudicots tested responded to streptol treatments with swollen and stunted roots. Within the *Gentianales* order, the seeds of some *Rubiaceae* and of *Psychotria capensis*, which does not belong to the nodulated *Psychotria* clade, failed at growing in the presence of the inhibitor. Thus far, *P. kirkii* is the only seed plant found to be insensitive to streptol (Fig. 5). We propose that the cyclitol specifically inhibits the biogenesis or remodelling of cell wall constituents during the elongation of root cells. However, a quick survey of the various polysaccharides present in the species included in our assays did not readily allow the identification of a cell wall polymer that would be unambiguously associated with the observed streptol-induced defects (Fig. 5).

## Discussion

This report describes the first natural cyclitol that specifically affects root growth of seed plants through cell wall disorganisation. The strong in vivo activity of (+)-streptol against potential plant competitors of nodulated *Psychotria* is congruent with the concept of allelopathy. Although difficult to demonstrate, the production of streptol by the bacterial symbionts may provide a fitness benefit to the host plant in the competition for space and nutrients. An α-d-glucose analogue disrupting cell wall integrity in growing roots would not only hamper water and nutrient uptake, but also increase the susceptibility to pests^68^. However, the ongoing adaptive role of the symbiosis is challenged by the recent placement of two non-nodulated *Psychotria* species within the nodulated clade^69^ and the pseudogenization of cyclitol biosynthetic genes in several symbionts^10^, indicating that cyclitol production is not the only contribution to the obligate character of the symbiotic association. In a current scenario^9^, climate change events of the Miocene resulted in arid conditions that compelled the rainforest to survive into restricted, hence highly competitive habitats. Remarkably, with the exception of *P. eminiana*, extant savannah-adapted African *Psychotria* associate with nodulating *Burkholderia* symbionts^69,70^. It would be pertinent to monitor the presence of cyclitols in the leaves of non-nodulating symbiotic *Rubiaceae*, where the bacterial symbionts invade the mesophyll, including several African species of the genera *Fadogia*, *Vangueria*, and about 20 *Psychotria* that do not belong to the nodulated clade^2,71^. The production of allopathic compounds may extend to other plant families and the acquisition of bacteria producing novel secondary metabolites may form a common basis for the establishment of plant–microbe symbioses.

(+)-Streptol causes intriguing root growth inhibition that can be mitigated by exogenous carbohydrate supply. The contrast between the structural CMT and cell wall defects observed in elongating root cells and the intact germination and aerial organs growth in (+)-streptol conditions might indicate a specific alteration of carbon partitioning into the EZ extracellular matrix. The complexity of plant cell wall biogenesis, that involves numerous enzymes, metabolic intermediates and dynamic membrane trafficking is not yet fully resolved^72^. However, the tightly controlled incorporation of monosaccharides into the primary cell wall of actively growing sink organs is central to plant life. Given its analogy to α-d-glucose, it is likely that (+)-streptol interferes with key glucose processing enzymes. A handful of mutations in such genes, metabolically upstream of CSCs or hemicellulosic and pectic synthesis machineries were reported to impact wall organisation to various degrees. The *Arabidopsis cinv1 cinv2* double mutant^64^ is impaired in sucrose-derived UDP‐glucose generation, which is required for cellulose synthesis, resulting in anomalous cellulose patterning and reduced anisotropy^62^. The *ugp1 ugp2* double mutant, which is devoid of UDP-glucose pyrophosphorylase activity, displays a drastic reduction in UDP-glucose and severe growth defects that can be rescued by exogenous sucrose supply^73^. Despite lower contents in downstream nucleotide-sugars and down-regulation of discrete CesAs transcription, *ugp1 ugp2* was, however, not reported to display prominent cell wall defects. Interestingly, cytosolic phosphoglucomutase *pgm2 pgm3* double mutants accumulate glucose-6-phosphate and only display mild alterations of the cell wall matrix, while the starch content increases^74^. Cytosolic UDP-glucose is therefore pivotal to the carbon channelling required for proper wall biosynthesis, as most nucleotide-sugars depend on its multi-step conversion to reach the Golgi synthetic routes to hemicelluloses and pectins. Further investigations of nucleotide sugar metabolism in *Arabidopsis* will be required to elucidate (+)-streptol specificity. Though the tolerance of *P. kirkii* towards the cyclitol remains enigmatic, we are currently investigating the genomes of *P kirkii* and non-nodulating sister species in search for possible (+)-streptol resistance mechanisms.

It is surprising that carbasugars displaying potent and specific activity against carbohydrate-active enzymes were so far rarely exploited in cell wall research. These compounds have several advantages: they are often non-metabolizable, do not undergo isomerization and show transition state analogy to virtually all natural substrates. We foresee (+)-streptol as a novel in vivo pharmacological tool to dissect plant cell anisotropic expansion beyond the well-characterized mechanisms of cellulose deposition.

The most exciting perspective of the natural product (+)-streptol, however, is its herbicidal potential, which may open novel strategies for sustainable agricultural management.

## Methods

### Preparation of *P. kirkii* extracts and leachates

Crude extracts were obtained from 100 g of fresh, 70% EtOH- and 1% hypochlorite surface-cleaned symbiotic *P. kirkii* leaves ground in liquid N_2_ and extracted with 1 l methanol:H_2_O (80:20) for 24 h in the dark. Solvent extract was filtered, mixed with 1 volume of chloroform and the methanol fraction was collected with a separatory funnel and lyophilized using a rotary evaporator under vacuum. The resulting 3.5–4 g viscous residual was dissolved in 12 ml pure H_2_O. Water extracts were obtained by reducing washed *P. kirkii* tissues (roots, 10 g; shoots, fruit flesh and flowers, 2 g) to powder in liquid N_2_, macerated 2 h in pure H_2_O and centrifuged. Resulting supernatants were filtered with 0.22 µm polyethersulfone filters (Millipore). *P. kirkii* leachates were obtained by bathing 100 g fresh, surface-cleaned leaves into 1 l sterile tap water for 48 h at room temperature. Leachates were filter-sterilized prior to in vitro use.

### Growth media and chemicals

The synthesis of (+)-streptol, (+)-streptol-β-glucoside, (+)-streptol-α-glucoside, (−)-streptol, (−)-streptol-β-glucoside, (−)-streptol-α-glucoside, and kirkamide was performed as previously described^16,17^. Unless otherwise stated, chemical inhibitors, hormones, dyes, reagents, culture media and supplements were purchased from Sigma-Aldrich, Switzerland. Isofagomine D-tartrate was purchased from Cayman Chemical, USA.

### Quantification of cellulose

Briefly, 5-day old Col-0 wildtype Arabidopsis seedlings grown on ½ MS 1% sucrose were transferred and vertically aligned onto fresh ½ MS plates without sucrose in presence of absence of 10 µM (+)-streptol, root tip positions marked, and let further grow for 48 h. About 20 mg primary root material per sample was collected from roots growing past the marking and snap-frozen in LN2. Cell wall isolation and crystalline cellulose content quantification was performed as described earlier^75^.

### Quantification of streptol and streptol glucoside by UHPLC-MS

The concentration of streptol and streptol glucoside was quantified using two different analytical methods using a UHPLC system (Ultimate 3000 LC, Thermo Fisher Scientific) coupled to a mass spectrometer (TSQ Quantum Ultra, Thermo Fisher Scientific). The first method consisted on analysing the samples using a Rezex RPM-monosaccharide Pb + (300 × 7.8 mm, Phenomenex) column, 100% H_2_O as eluent, a column oven set-up at 80 °C, and a flow at 0.45 µl min^−1^. The detection was achieved in positive mode by single ion monitoring (SIM) using the mass of 194 Da for streptol (M + NH_3_ + H)^+^ and 361 Da for streptol glucoside (M + Na)^+^. A calibration curve was built with the following solutions: 0.5, 1, 5, 10 and 50 µg ml^−1^ for streptol glucoside and 0.5, 1, 5 and 10 µg ml^−1^ for streptol and 4 µl were injected. The extracts from *Psychotria kirkii* and streptol glucoside-treated *Arabidopsis,* and *Arabidopsis* controls were prepared at 3 mg ml^−1^ and 4 µl were injected. The second method consisted on analysing the samples using a BEH amide (2.1 × 100 mm, Waters) column, a solvent system composed of MeCN:H_2_O (A, 8:2, 0.1% NH_4_OAc) and MeCN:H_2_O (B, 2:8, 0.1% NH_4_OAc), a column oven set-up at 35 °C, and a flow of 0.2 ml min^−1^. The gradient varied from 0 to 50% of B in 7 min, 50% to 100% of B in 0.05 min and the column was washed with 100% of B for 2 min. Streptol has a retention time of 4.0 min and was detected in negative mode by single reaction monitoring (SRM) using the specific fragmentation of the protonated molecule [M–H]^–^ at *m*/*z* 175 Da into the fragment ion at *m*/*z* 121 at a collision energy of 19 eV. Streptol glucoside had a retention time of 5.3 min and was detected in negative mode by SRM using the specific fragmentation of the protonated molecule [M–H]^–^ at *m*/*z* 337 Da into the fragment ion at *m*/*z* 139 at a collision energy of 16 eV. A calibration curve was built with the following solutions: 0.1, 0.5, 1, 5 and 10 µg ml^−1^ for streptol glucoside and streptol and 4 µl of this solution were injected. Gelose extracts from symbiotic and aposymbiotic *P. kirkii* spent media were prepared at 15 mg ml^−1^, Arabidopsis root and shoots samples were prepared at 3 mg ml^−1^. Calibration curves are available in the “Supplementary Information S1” material.

### Plant material

#### Arabidopsis lines

*Arabidopsis thaliana* (L.) Heynh. var. Columbia (Col-0) was used as wild-type strain. Twenty other *A. thaliana* ecotypes including *Ler1* and *Was0* were tested against *P. kirkii* extracts and pure (+)-streptol with similar results. The single Arabidopsis mutants *cwinv1* (AT3G13790)*, cwinv2* (AT3G52600), *cwinv3* (AT1G55120), *cwinv4* (AT2G36190), *cwinv5* (AT3G13784), *cwinv6* (AT5G11920), *cinv1-4* (AT1G35580), *cinv1-8 (*AT1G35580), *cinv2* (AT4G09510), *stp1-1* (AT1G11260), *stp13-1* (AT5G26340) and the double mutants *stp1 stp13* and *stp1 stp4* were kindly provided by Diana Santelia (ETH Zurich, Switzerland); *prc1* (AT5G64740)*, cev1* (AT5G05170) and *rsw1* (AT4G32410) were obtained from Clara Sanchez-Rodriguez (ETH Zurich, Switzerland). The Arabidopsis pCYCB1;1::DB‐GUS and pUBQ10::EYFP-TUB6 fusion constructs were kindly provided by Joop Vermeer (University of Neuchâtel, Switzerland). Other lines used in this study were maintained in our laboratory.

Other plant species used in this study are listed in Supplementary Table 1. Non-commercial seeds were kindly provided by Peter Enz (University of Zurich’s Botanical Garden) with relevant permissions or ordered with relevant permissions from the SAG (Goettingen University, Germany) and the IMSC (University of Freiburg, Germany). All the experimental work on plant material described in this study complies with the relevant institutional, national, and international guidelines and legislation.

### Plants culture conditions and growth inhibition assays

Unless otherwise stated, all plant species were grown in a Conviron growth cabinet with 16/8 h, 20/18 °C day/night cycles, 60% relative air humidity and a photon flux of 100 μmol m^−2^ s^−1^ during light periods.

#### Arabidopsis assays

*Arabidopsis* seeds were surface-sterilized and kept for imbibition in the dark at 4 °C for at least 24 h. Long term growth experiments were carried out with seeds vertically grown on half strength Murashige and Skoog (½MS, pH 5.7) medium 1.5% agar with or without 1% sucrose supplement. For time-lapse series, square plates were mounted on a vertical Epson Perfection 1240U flatbed scanner adapted with a 100 μmol m^−2^ s^−1^ white LED light source (16/8 h day/night cycles) and growth was recorded every 4 h using the VueScan 9.4.32 software (Hamrick Software, USA; https://www.hamrick.com). Short-term experiments were carried out in 96-well microtiter plates in sterile tap water. The pure inhibitors were supplemented at given concentrations. Adult Arabidopsis plants were grown in the same conditions in Magenta boxes onto 0.8% agar ½MS and supplemented with *P. kirkii* extracts at given concentrations. Arabidopsis root cells suspension cultures were obtained from 1 g finely-chopped, mature, axenically grown roots in ½MS 3% sucrose supplemented with 0.5 mg l^−1^ benzylaminopurine, 1 mg l^−1^ napthalene acetic acid, 1 mg l^−1^ indole acetic acid, 1 mg l^−1^ 2,4-dichloro-phenoxyacetic acid and maintained by subculturing at 4-week intervals. Fresh subcultures were grown for 2 weeks prior to 10 µM streptol treatment in the exponential growth phase. Growth and fitness of cells were frequently evaluated under a Leica DM600B dissecting microscope (Leica, Germany). Quantitative analysis of *Arabidopsis* root gravitropism was performed as a proxy for evaluating auxin distribution disturbances. Surface-sterilized seeds were grown vertically on ½MS 1% sucrose 1% agar for 5 days and were next vertically aligned onto fresh plates containing increasing doses of *P. kirkii* extracts and allowed further growth in the light for 2 h. Gravistimulation was achieved by tilting the plates to 90° and letting the seedlings grow for 24 h in the dark. Flatbed scanner acquisitions prior to and after stimulation were used to determine root growth and tip reorientation in the gravity field.

#### Allelopathy assays

Surface-sterilised lettuce seeds (*Lactuca sativa* var. acephala) and black mustard seeds (*Brassica nigra*) were treated after imbibition on 55 mm Petri dishes lined with sterile filter paper (Whatman, diameter 50 mm) soaked with 1 ml symbiotic *P. kirkii* crude extracts or filtered water extracts from roots, shoots, flower and seed flesh or *P. kirkii* leaf leachate and incubated in the dark at room temperature for 6 days. Using the same setting, lettuce seeds were grown in the vicinity of intact, surface-sterilized mature *P. kirkii* drupes or pyrenes. Control treatments were performed with sterile tap water. Seeds with radicles as long as seed diameter were considered germinated. For soil assays, black mustard seeds were sown after imbibition on individual peat-soil plastic pots (15 seeds each) and cultured in a growth cabinet as described for 10–15 days. Treatments consisted of either watering the pots with *P. kirkii* leachates, crude extract dilutions (0.1% v/v) or germinating the seeds on soil amended with mulched *P. kirkii* leaves (100 g leaves per litre of soil); tap water was used for controls. Pots were watered to their water holding capacity once per week. Successfully established seedlings were counted 7 days after sowing.

#### Other plant species

Algae were axenically cultured and maintained on K medium as indicated by the Culture Collection of Algae at the university of Göttingen, Germany (http://sagdb.uni-goettingen.de/culture_media/). Inhibitors treatments were performed in 2 ml liquid K medium in 6-well plates supplemented with 100 μl of *P. kirkii* crude extract or 10 µM streptol and incubated at 22 °C under continuous 30 μE illumination and shaking for a week. Growth and fitness of algal cells were frequently evaluated under a Leica DM600B dissecting microscope (Leica, Germany). Mosses were axenically cultured and maintained on solid BCD medium (1 mM MgSO_4_, 10 mM KNO_3_, 45 μM FeSO_4_, 1.8 mM KH_2_PO_4_ (pH 6.5 adjusted with KOH), 0.22 μM CuSO_4_, 0.19 μM ZnSO_4_, 10 μM H_3_BO_3_, 0.1 μM Na_2_MoO_4_, 2 μM MnCl_2_, 0.23 μM CoCl_2_, and 0.17 μM KI). *P. kirkii* crude extract (10% v/v) or 10 µM streptol treatments were performed in solid BCD 6-well plates from gametophytic tissues and incubated for 30 days. Growth and fitness of colonies were frequently evaluated and representative specimens documented under a Leica M165FC stereo microscope (Leica, Germany). About 100 mg dried fern spores were collected from fronds of a mature specimen through a 60 µm mesh, suspended in 2 ml ddH_2_O and sterilized with 2.5% v/v NaClO, 0.05% Tween for 5 min and washed five times with sterile ddH_2_O. Spores were spread on ½MS agar 6-well plates amended with the inhibitors at given concentrations, let for 15 h at RT under red-filtered light and cultivated under 65 µE white light (16 h day, 20 °C and 8 h night, 18 °C). Spermatophytes seeds were surface-sterilized with 2–10% v/v NaClO, 0.05% Tween and washed five times with sterile ddH_2_O. Gymnosperms seeds were kept in Parafilm-sealed Petri dishes lined with wet sterile filter paper in the dark at 4 °C at least 3 weeks before treatments. Seeds were treated after imbibition at 4 °C on 55 mm Petri dishes lined with sterile filter paper soaked with 1 ml crude extract (10% v/v) and let to germinate in the dark at 22 °C. Post germination, primary roots were inspected under a Leica M165FC stereo microscope. *Psychotria kirkii* adult plants are maintained in a dedicated greenhouse at the botanical garden of the University of Zurich. For cyclitols quantification in *P. kirkii* exudates, fresh surface-sterilized pyrenes were germinated and cultured in Magenta boxes onto 0.8% agar ½MS to the four leaves stage. Aposymbiotic plantlets were obtained by heating the seeds to 52 °C for 12 min in sterile water prior to germination.

Phenotypical measurements were performed with Fiji 1.53 (https://imagej.net) from flatbed scanner acquisitions (600–1200 dpi) or original micrographs. Curve fitting and statistical analysis were performed with the Prism 8 software (GraphPad, USA; https://www.graphpad.com).

### Histology and microscopy

Unless specified otherwise, samples were observed under a Leica DM5500Q confocal microscope fitted with a TCS SPE confocal unit (Leica, Germany), an ACSAPO 40× oil-immersion objective (NA = 1.15, Leica) or an ACSAPO 10× dry objective (NA = 0.3, Leica), and laser lines set at 405, 488, and 532 nm. The images were acquired using the LAS software (Leica, Germany; https://www.leica-microsystems.com).

In order to measure root cells dimensions, live Arabidopsis seedlings were stained for 2 min in 1 mg ml^−1^ propidium iodide, rinsed twice with tap water and optical sections of primary root apices were acquired with the confocal microscope. Measurements were performed with FiJi. Root, hypocotyl and inflorescence transversal semi-thin sections were obtained from 3% agarose-embedded samples. Deeper root tissue investigations were carried out using the modified pseudo-Schiff propidium iodide (mPS-PI) method. In short, whole seedlings were fixed in 50% methanol, 10% acetic acid at 4 °C for at least 12 h. Samples were transferred to 80 °C 80% ethanol for 5 min, transferred back to fixative for 1 h; rinsed with ddH_2_O and incubated in 1% periodic acid at room temperature for 40 min. Samples were rinsed with ddH2O and incubated in 100 mM Na_2_S_2_O_5_, 0.15 N HCl with 1 mg ml^−1^ propidium iodide for 2 h. Samples were transferred onto microscope slides and covered overnight with a chloral hydrate solution (4 g chloral hydrate, 1 ml glycerol, and 2 ml H_2_O). Excess chloral hydrate was removed and samples mounted in Hoyer's solution (30 g gum arabic, 200 g chloral hydrate, 20 g glycerol, and 50 ml H_2_O). Images were acquired with under the confocal microscope.

Other cell wall-related histochemical staining procedures were essentially performed as described^76^. For epidermal cellulose microfibrils visualisation, whole seedlings were stained with 1 mg ml^−1^ Direct Red 23 in sterile tap water for 30 min, washed thrice and mounted in water. Image acquisition was performed under the confocal microscope.

To qualitatively assess cytokinesis, nuclear staining and cell wall counterstaining of 5-day old *Arabidopsis* seedlings grown in presence or absence of 5 μM streptol was achieved with either 2.5 μM SYTO 9 (Invitrogen, USA) and 5 μM propidium iodide or with 10 μg ml^−1^ DAPI (0.1% Triton-X100, Thermo Fisher Scientific, USA) and 1 mg ml^−1^ Direct Red 23 for 20 min in sterile tap water. The presence of polyploid cells or cell plate stubs was monitored under the confocal microscope (20 plants per treatment).

In order to visualize and quantify cortical microtubules orientations, pUBQ10::EYFP-TUB6 signals from 5-day old seedlings grown in presence or absence of 5 μM streptol were acquired in 0.5–1 μm step z-stacks under a the confocal microscope throughout outer epidermal surfaces. Maximum z-projections were analysed with FibriTool^77^.

DR5::GFP signals in Arabidopsis were imaged under the confocal microscope after transferring 5 days old seedlings onto the given inhibitor concentrations for 24 h. Histochemical GUS staining was performed on whole pCYCB1;1::DB-GUS seedlings essentially as described^78^, fixed in 96% ethanol and mounted in chloral hydrate.

#### Electron microscopy

Whole seedlings were chemically fixed with 2.5% glutaraldehyde in 0.1 M cacodylate buffer, rinsed with pure water, excess liquid removed, plunged-frozen in liquid propane mounted on a 3.05 mm diameter 400 mesh copper grid and stored in liquid N_2_. Samples then were mounted on a grid holder and freeze-dried at − 95 °C for 1 h at 10^–6^ mbar vacuum. Samples were quickly transferred to a Zeiss Auriga 40 CrossBeam focused ion beam-scanning electron microscope (Zeiss, Germany) to check preparation quality. Samples were then further dried at − 95 °C for 1 h, then coated with 2.5 nm of Pt/C moving the gun between 0° and 45° and rotating the stage at 40 rpm plus 2.5 nm Pt/C double axis rotary shadowing. Samples then were again transferred to the SEM using a Leica VCT100 cryo transfer system (Leica, Germany) and imaged at − 112 °C, at 5 kV; Inlens, 1.4 and 5 mm WD. Preferred orientation of root cell wall structures from SEM acquisitions were inferred by using the FiJi Directionality analysis v2.3.0 plugin applying the Fourier components analysis method and a 90 bins partition.

### Microbial culture conditions and growth inhibition assays

#### Bacteria

*Escherichia coli* ATCC25922, Burkholderia *cenocepacia* H111, *Bacillus subtilis*, *Staphylococcus epidermidis* RP62a and *Pseudomonas aeruginosa* PAO1 were cultured in lysogeny broth (LB) at 30 or 37 °C until reaching the exponential growth phase and cell density adjusted to OD_600nm_ = 0.1 in sterile saline for downward experiments. Growth curves in the presence of inhibitors were obtained in a Sirius HT 96-well microtiter plate reader (BioTek Instruments, Switzerland) at 37 °C with constant shaking in a final volume of 100 µl. Mucoid exopolysaccharide production was assessed by spotting bacterial suspensions onto yeast extract medium (0.5 g l^−1^ yeast extract, 4 g l^−1^ mannitol, 15 g l^−1^ agar) gradient plates with the given inhibitor concentration as the highest dose. Biofilm biomass was evaluated in the presence of inhibitors by growing 100 μl of cell cultures to the late log phase in ABC medium in sealed 96-well polystyrene microtitre plates for 72 h at 28 °C; Abs_550nm_ was measured. Growth medium was removed and 100 μl of a 1% (w/v) aqueous solution of crystal violet added at room temperature for 30 min; excess of dye was removed and wells thoroughly washed and dried. Crystal violet was solubilized in 120 μl DMSO and Abs_570nm_ measured. The Biofilm Index (BI) was normalized with respect to growth as BI = Abs_570nm_/Abs_550nm_.

#### Fungi and oomycetes

Agar plugs of actively growing *Fusarium solanii, Rhizoctonia solanii and Botrytis cinerea* were transferred to the borders of fresh malt extract 2% agar plates. Sterile paper disks were impregnated with the inhibitors at given concentrations and placed in the middle of the plates and incubated in the dark at 22 °C for 48 h. Mycelial growth was monitored. Approximatively 10^5^ spores per ml harvested from mature *Fusarium solanii* and *Botrytis cinerea* as well as hyphal cuttings from *Rhizoctonia solanii* were placed in 6-well plates with sterile tap water supplemented with the given inhibitors and incubated for 24 h in the dark at 22 °C with gentle shaking (110 rpm). Germination of spores and regeneration of cut hyphae was evaluated under a Leica DM600B epifluorescence microscope after 100 μg ml^−1^ Calcofluor White staining. *Phytophthora infestans* Rec01 was maintained on V8 medium in our laboratory. Agar plugs of actively growing *P. infestans* were transferred to the middle of fresh rye 1.5% agar plates and 8 mm wells produced with a cork borer were filled with *P. kirkii* crude extracts or sterile water as solvent controls. Mycelial growth was monitored. Mature sporangia were harvested with sterile H_2_O from 10 days mycelia, filtered through sterile cheese cloth and inhibitors were supplemented at the given concentrations. Sporangia germination was stimulated by vigorous vortexing and let to germinate for in the dark at 22 °C for 16 h. Spore germination was assessed under a Leica M165FC stereo microscope and germ tube length was measured from Leica DM600B dissecting microscope brightfield micrographs using ImageJ software.

### Cytotoxicity of (+)-streptol on animal cell line

#### Brine shrimps

*Artemia salina* were hatched from dehydrated eggs (Artemio Pur, JBL GmbH Germany) under constant shaking and 60 µE white light illumination at room temperature in artificial sea water (NaCl 37 g/l, pH 8.5 NaOH) supplemented with the given concentrations of inhibitors. After 72 h, the different stages of development of individuals were assessed under a Leica M165FC stereo microscope. No differences in nauplii swimming motility were observed between treatments.

#### Human cells

Human colon Duke's type B adenocarcinoma LS 174 T cell line was purchased from the Deutsche Sammlung von Mikroorganismen und Zellkulturen (ACC 759) and the cells were grown in Dulbecco's Modified Eagle Medium (DMEM) with 10% heat-inactivated Fetal Bovine Serum (FBS), 0.1% penicillin–streptomycin in an incubator (37 °C, 5% CO_2_, 95% humidity). LS 174 T cells (4 × 10^3^) were seeded in 96-well cell culture plates in a final volume of 100 µl and allowed to attach for 24 h in the incubator. To evaluate the cytotoxic potential of (+)-streptol, LS 174 T cells were treated with increasing concentrations of the compound in DMEM medium with 10% heat-inactivated FBS, 0.1% penicillin–streptomycin for 5 days in the same growth conditions. Cytotoxicity was determined by replacing the medium with 100 µl of DMEM containing 10% colorimetric Cell Counting Kit-8 (Dojindo Molecular Technologies, Inc.), incubating the cells for another 90 min, and determining dehydrogenase activities by measuring the absorbance at 450 nm following the manufacturer’s instructions. Absorbance at 650 nm was also measured and the value was subtracted to samples’ values as the background. Cells in medium were used as negative control and wells without cells were set as blank.

All photographs displayed in this work were acquired from a Nikon D90 digital camera equipped with a NIKKOR AF-S Micro 60 mm f/2.8G ED macroobjective.

## Supplementary Information

Supplementary Information.

## Data Availability

The data supporting the findings reported in this work are available within the manuscript and the associated Supplementary Information files and from the corresponding authors upon reasonable request.
