# Supplementary material for: Leaf nodule endosymbiotic Burkholderia confer targeted allelopathy to their Psychotria hosts
Source: Sci Rep. 2021 Nov 17;11:22465. doi: 10.1038/s41598-021-01867-2 (PMC8599487; doi:10.1038/s41598-021-01867-2)
Supplement: Supplementary file 1 — Supplementary Information. [file 41598_2021_1867_MOESM1_ESM.pdf]

Supplementary Information to

Leaf-nodule endosymbiotic *Burkholderia* confer targeted allelopathy to their *Psychotria* hosts

Antri Georgiou, Simon Sieber, Chien-Chi Hsiao, Tatyana Grayfer, Jacob L. Gorenflos López, Karl Gademann, Leo Eberl, and Aurélien Bailly.

Supplementary figures

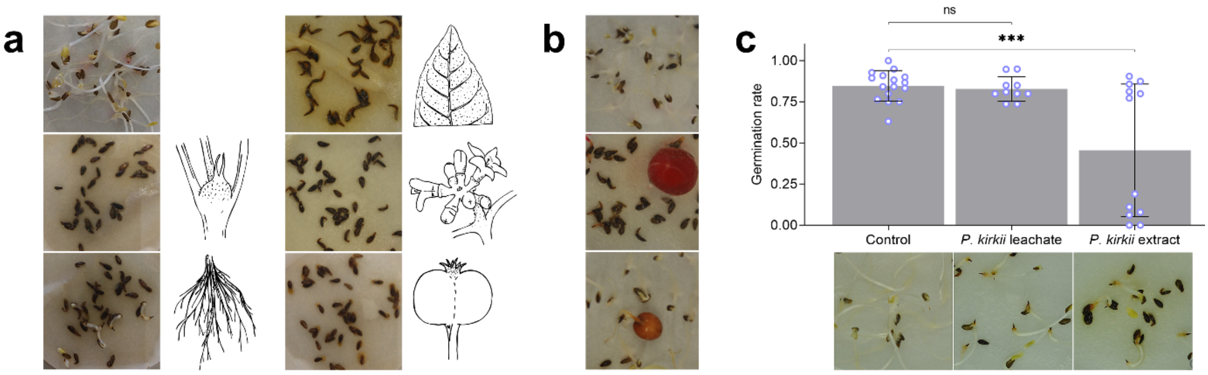

Supplementary Figure 1: *P. kirkii* extracts contain lettuce root-inhibiting activity

(**a**) *P. kirkii* crude water extracts from apical shoots, roots, leaves, flowers and fruits contain strong lettuce root inhibitors. Representative results are displayed. Three independent experiments with three biological replicates were performed for each treatment. Top left image represents water controls. (**b**) Intact *P. kirkii* mature drupes laid together with lettuce seeds in Petri dishes are sufficient to prevent root growth. Pyrenes alone did not display lettuce root inhibition properties. Five biological replicates were performed for each treatment. Top image represents water controls. (**c**) *In vitro* lettuce seeds germination rates in presence of *P. kirkii* leachates or leaf extracts. Significant differences between treatments and water control were analysed in a one-way ANOVA with Dunnett's post-hoc test (*** p-value < 0.001; ns, not significant). Values represent the mean values of the given biological replicates (20 seeds per replicate); circles indicate data distribution, standard deviations are indicated. Representative results are displayed below the corresponding treatment.

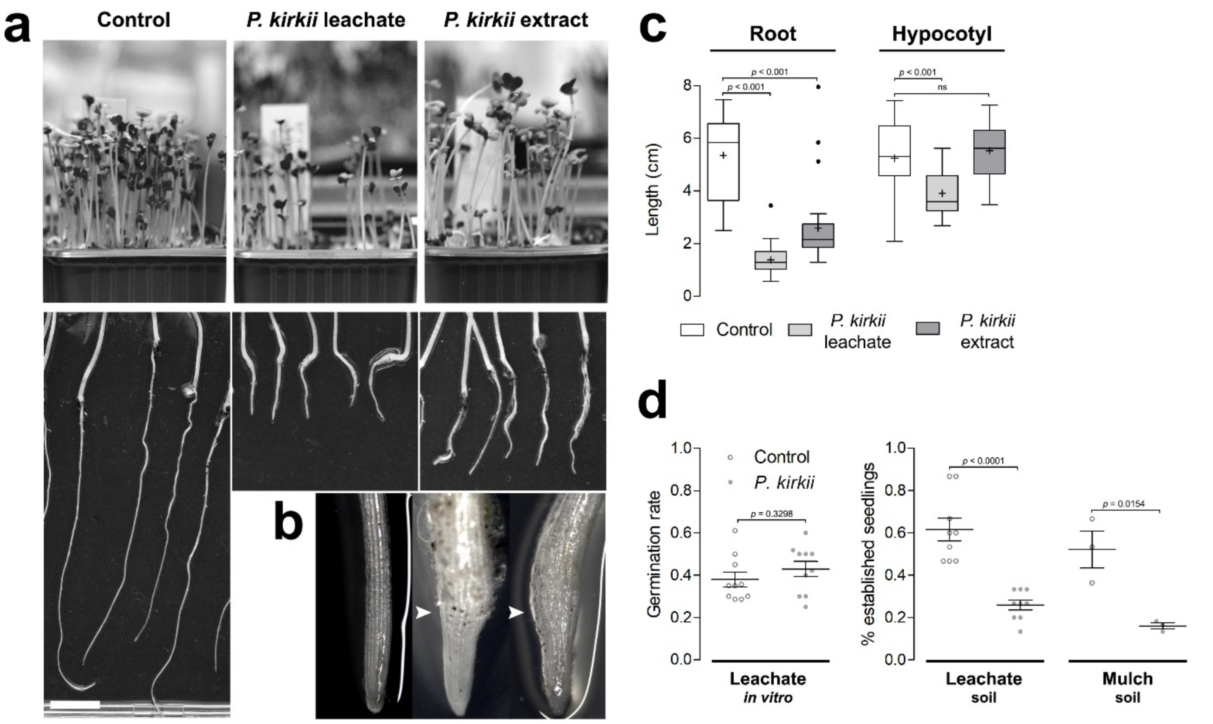

Supplementary Figure 2: *P. kirkii* extracts exert *bona fide* allelopathic effects on *Brassica nigra* seedlings

(**a**) Root development of *B. nigra* seeds on soil is arrested by *P. kirkii* leachates and extracts supplementation. Washed roots were aligned on water-agar plates for measurements. Three independent experiments with three biological replicates were performed for each treatment. Representative pictures are shown. Bar = 1 cm. (**b**) Higher magnification of the *B. nigra* seedlings root apices. From left to right: water control, *P. kirkii* leachate, *P. kirkii* extract. Arrowheads point to the swollen region of the root. (**c**) Box plots of root and hypocotyl lengths of the treated *B. nigra* seedlings; + signs represent the mean values, dots outliers. The data of one experiment is shown (n = 30). Significant differences between treatments and water control were analysed in a one-way ANOVA with Dunnett's post-hoc test (p-values are indicated; ns, not significant).(**d**) Left, *in vitro* germination rates of *B. nigra* in the presence of *P. kirkii* leachates. Significant differences between treatments and water control were analysed in a two-sided unpaired t-test. Values represent the mean values of 10 biological replicates (20 seeds per replicate); circles indicate data distribution, standard errors are indicated. Right, *B. nigra* seedlings establishment in treated soils. Significant differences between treatments and water control were analysed in a two-sided unpaired t-test. Values represent the mean values of the given biological replicates (15 seeds per replicate); circles indicate data distribution, standard errors are indicated.

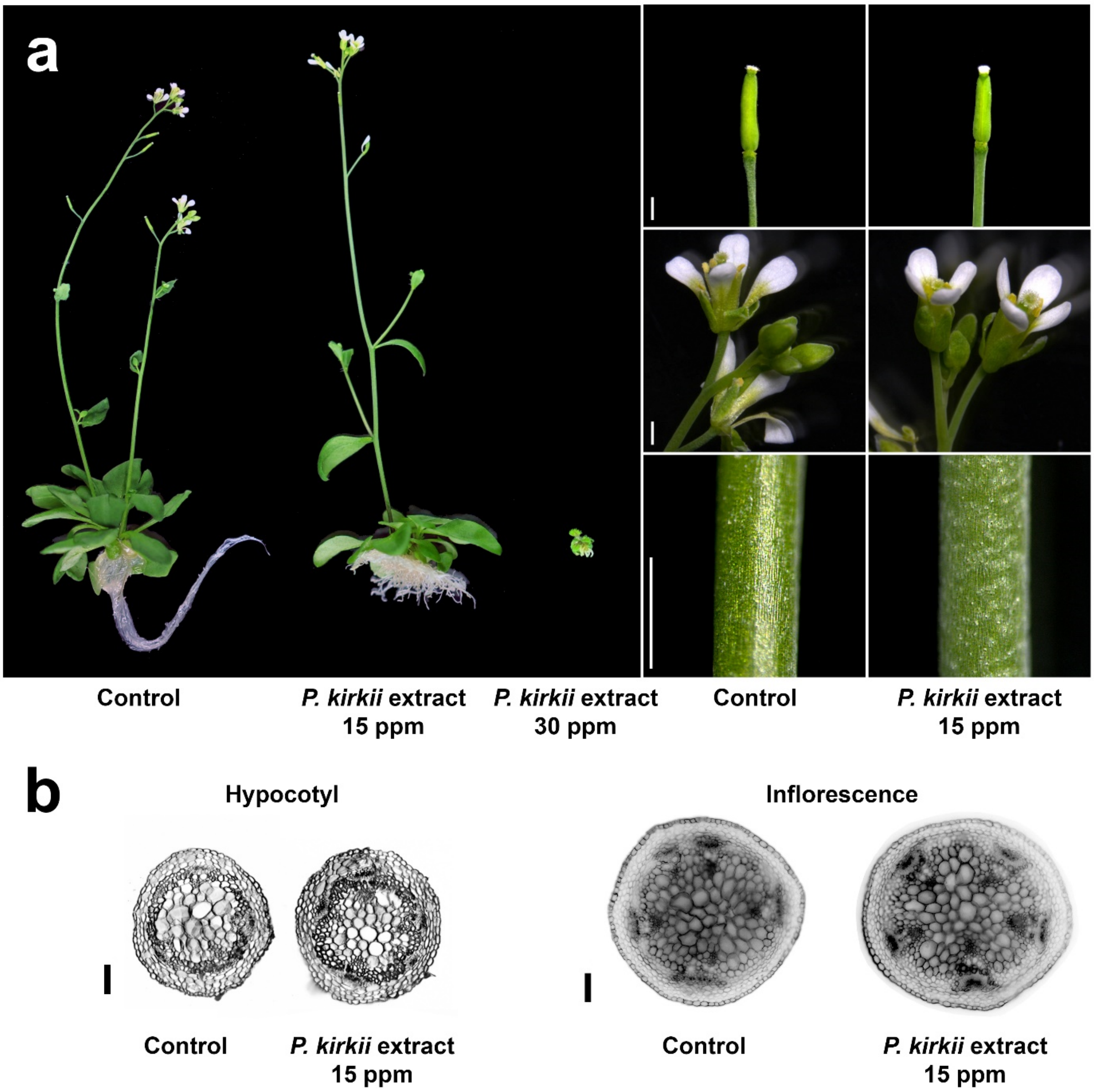

Supplementary Figure 3: Arabidopsis aerial organs fully develop on root growth-restrictive *P. kirkii* extracts

(**a**) Left, 60 days-old representative plants at given treatments. Right, magnification of siliques, flowers and inflorescences. Bar = 1 mm. (**b**) Representative semi-thin transversal sections through the mid-part of hypocotyls and first inflorescence internode. Cell walls were stained with Calcofluor White. Bar = 100 µm.

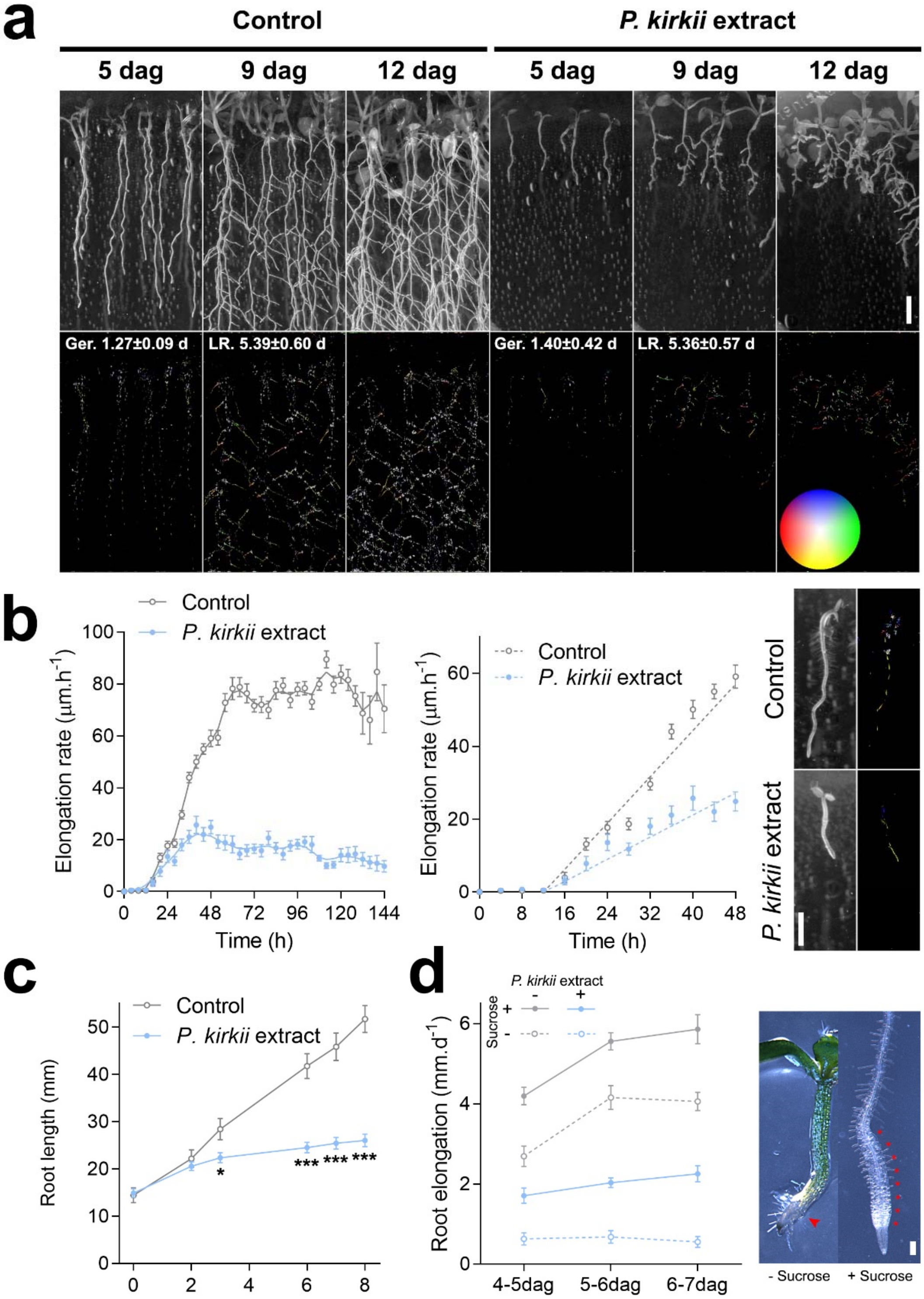

Supplementary Figure 4: *P. kirkii* extracts affect root growth but not morphogenesis and development

(**a**) Time lapse of *Arabidopsis* root growth at 4h intervals. Lower panels represent flowJ-generated gradient-based optical flow estimations in the following developmental time frames: 0-5 dag, primary root growth; 5-9 dag, lateral root emergence and growth; 9-12 dag, further root system development. Ger., day of germination with standard deviation. LR., day of first lateral root emergence with standard deviation. No significant differences between treatment (n = 14) and untreated control (n = 30) were

found in a two-sided unpaired t-test. Disc displays the DC index. Bar = 1 cm. (**b**) Left, *Arabidopsis* primary root elongation rates in presence of *P. kirkii* extracts. Middle, the same data, first 48 hours. Dashed lines represent linear regression from effective germination. Right, representative seedlings at 2 dag with gradient-based optical flow estimations. Bar = 1 mm. (**c**) Growth of control-grown *Arabidopsis* roots transferred to *P. kirkii* extracts conditions. (**d**) Left, *P. kirkii* extracts-mediated *Arabidopsis* primary root growth inhibition is mitigated by sucrose supplementation. Right, representative seedlings in presence of *P. kirkii* extracts with or without sucrose. Arrowhead indicates the root-shoot junction. Asterisks mark the swollen root tissue above the meristem. Bar = 100 µm. dag = days after germination.

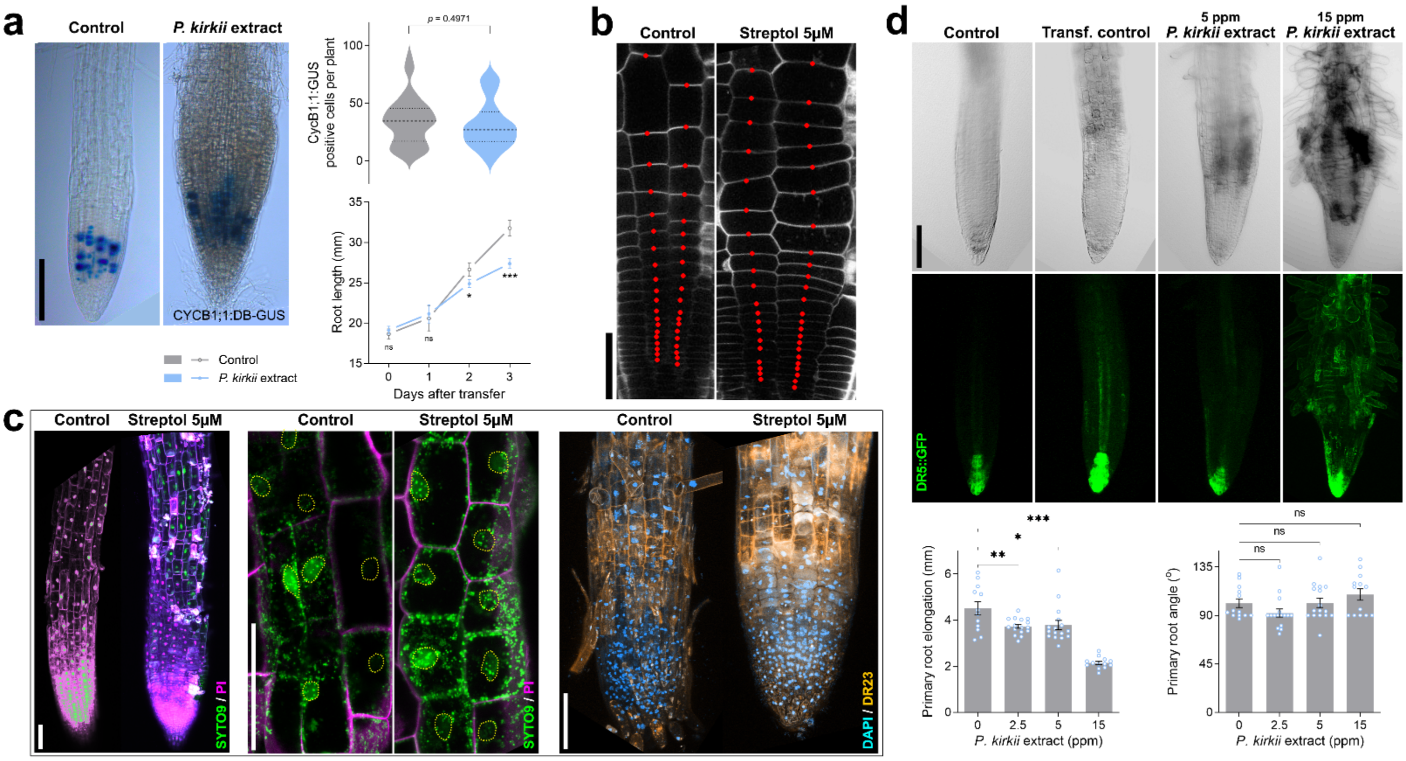

Supplementary Figure 5: *P. kirkii* extracts do not affect cytokinesis nor auxin distribution

(**a**) Left, expression pattern of the cell cycle marker pCYCB1;1::DB-GUS in presence and absence of *P .kirkii* extracts. Representative seedlings. Bar = 100 µm. Right, lower panel displays the root inhibition of tested seedlings. Significant differences between treatment and untreated control for each timepoint were analysed in a two-sided unpaired t-test (* *p*-value < 0.05; *** *p*-value < 0.001; ns, not significant). Values represent the mean values of three biological replicates (n = 10 plants each); standard errors are indicated. Right, upper panel, violin plots of the number of root GUS-positive cells per plant. Significant differences between treatment and untreated control were analysed in a two-sided unpaired t-test (p-value is indicated). Data distribution of four biological replicates (n = 10 plants each). (**b**) Tangential longitudinal optical sections of mPS-PI-stained epidermal layers in the presence or absence of streptol. Note that symptomatic cells display completed primary cell walls at the equator. Red dots indicate the centre of each wall in the longitudinal axis. Representative CLSM single plane acquisitions. Bar = 50 µm. (**c**) Left, SYTO9/Propidium Iodide double staining of 5-day-old seedlings in the presence or absence of streptol. Note the single nuclei and completed wall biogenesis in symptomatic plants cell files. Middle, magnification in the EZ; nuclei are delineated by yellow dots. Right, DAPI/DR23 double staining present similar nuclei distribution and wall formation in streptol and control conditions. Maximum projections of CLSM z-stacks. Bars = 50 µm. (**d**) Upper panel, DR5::GFP signal patterns are unaltered in *P. kirkii* extracts conditions. Representative light micrographs and corresponding maximum projections of DR5::GFP signal CLSM optical sections of the given treatments. Bar = 100 µm. Lower panel, primary root elongation and tip reorientation after a 24h 90°-gravistimulation. Note that, despite root growth inhibition, gravitropic response was intact in all treatments. Significant differences between treatments and water control were analysed in a one-way ANOVA with Dunnett's post-hoc test (* *p*-value < 0.05; ** *p*-value < 0.01; *** *p*-value < 0.001; ns, not significant). Data represent mean values; circles indicate data distribution, standard errors are indicated.

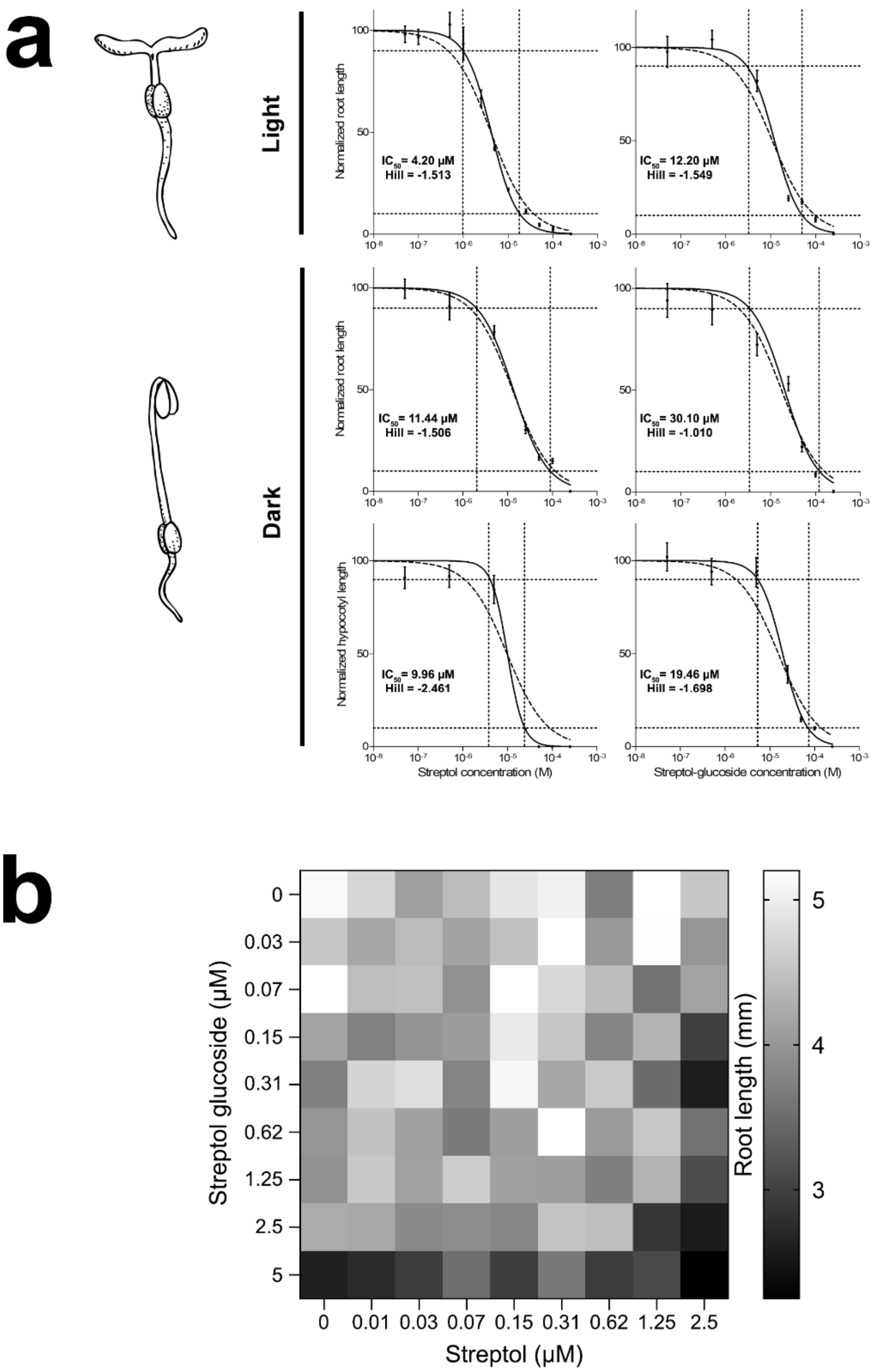

Supplementary Figure 6: Streptol and streptol glucoside similarly inhibit *Arabidopsis* root growth

(**a**) Dose-response curves of *Arabidopsis* root and hypocotyl growth to streptol and SG during photo- and skotomorphogenesis. Data represent normalized mean values of three biological replicates (8 plants per replicate); standard errors are indicated. Curves show log(inhibitor) vs. normalized response non-linear fitting with (apparent) variable Hill slope (solid lines) or Hill slope = -1 (dashed lines). Best-fitted apparent IC_{50} and Hill coefficient values are indicated. Dotted grid lines mark the 10% and 90% inhibition and corresponding concentrations. (**b**) Streptol vs SG checkerboard root growth assay at sub-IC_{50} concentrations. Data represent mean values of two biological replicates.

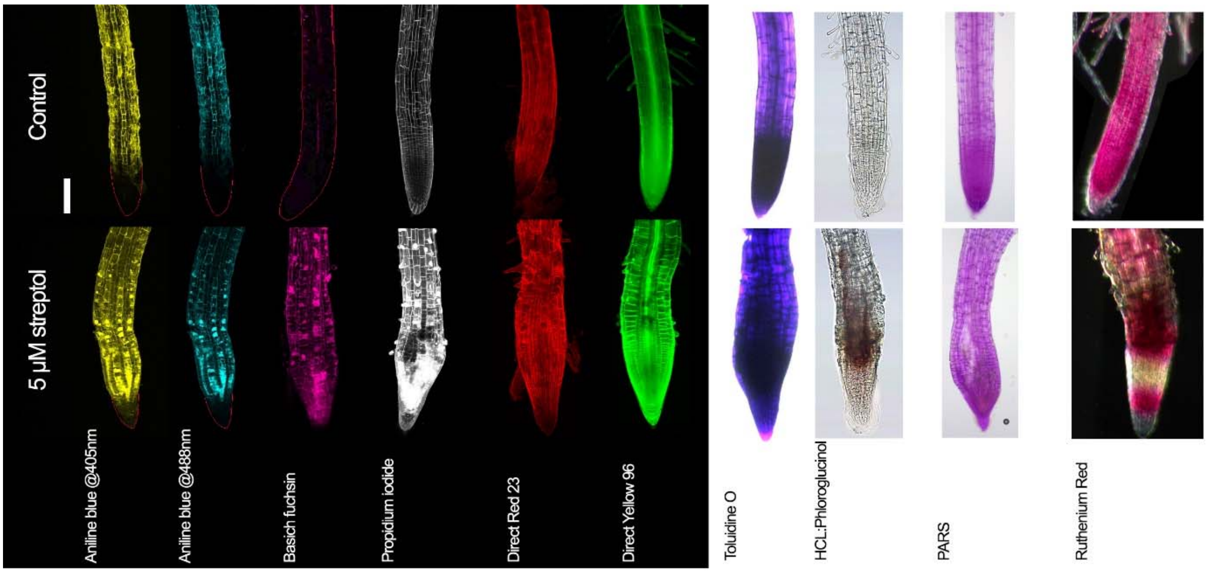

Supplementary Figure 7: Cell wall histochemistry of streptol-treated *Arabidopsis* roots

Representative light or CLSM micrographs of the given histochemical staining techniques. For each, treatment and corresponding untreated control data were acquired using identical acquisition settings. Bar = 100 µm.

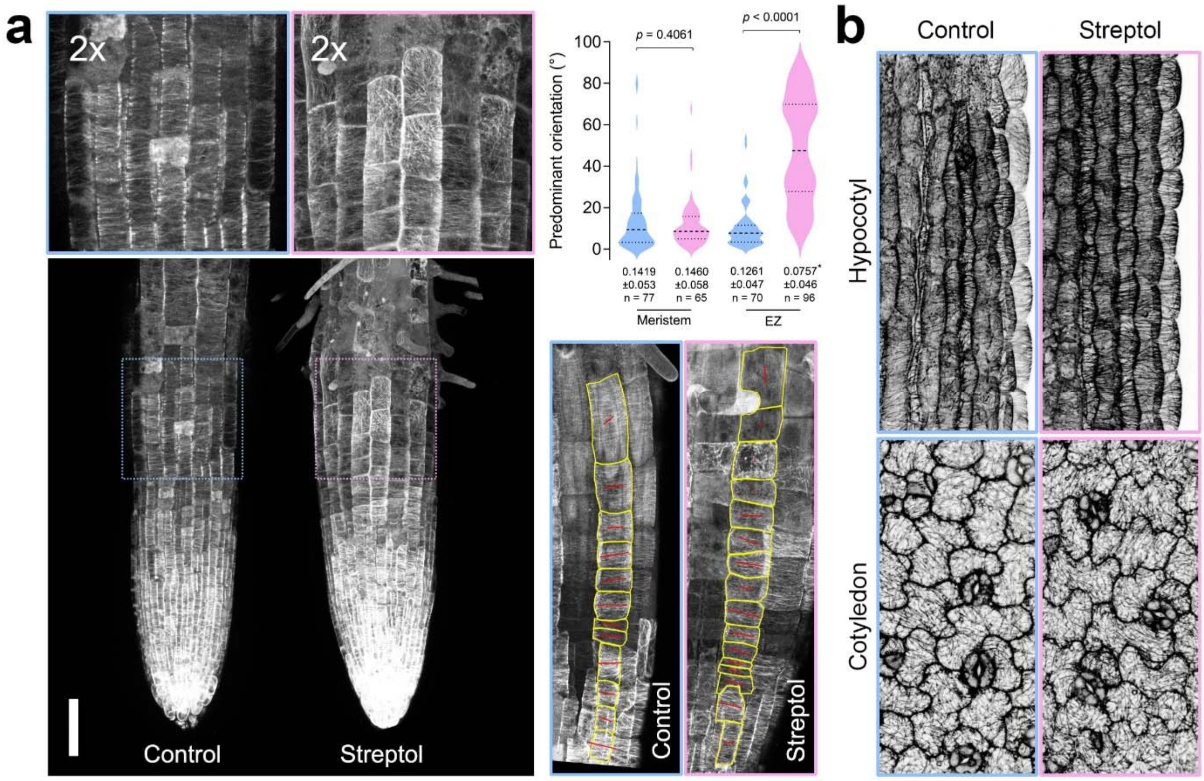

Supplementary Figure 8: Streptol treatment disorganizes cortical microtubules in the root elongation zone.

(**a**) Left, overview of pUBQ10::EYFP-TUB6 signal distribution in the root apex epidermis in presence and absence of streptol. Composite CLSM micrographs maximum z-projections. Bar = 50 µm. Right, upper panel, FibriTool quantitative analysis of the anisotropy of CMT arrays. The orientation angle of fibres orthogonal to the longitudinal axis is represented. Significant differences between treatment and water control were analysed in a two-sided unpaired t-test. Values represent the mean values of 6 randomly chosen individual plants; *p*-values are indicated; *n* indicates the total number of cells analysed; numbers represent anisotropy and standard deviations. Significant differences between treatment and water control were analysed in a two-sided unpaired t-test (* *p*-value < 0.0001). Lower panel shows representative whole cell FibriTool outputs during transition to the fast elongation zone. Yellow lines represent the analysed ROIs, red lines visualize average orientation and anisotropy score. Line length was set to 5. (**b**) Z-maximum projections of pUBQ10::EYFP-TUB6 signals in the epidermal layers of *Arabidopsis* hypocotyls and cotyledons in presence or absence of streptol.

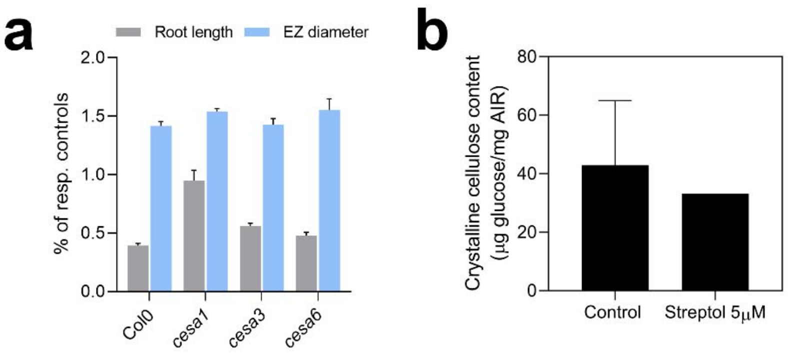

Supplementary Figure 9: Response of *Arabidopsis* CesA mutants to 5 µM streptol

(**a**) Response of *Arabidopsis* CesA mutants to 5 µM streptol. Root length and root EZ diameter relative to respective untreated controls values are given. Data represent mean values, standard errors are indicated (n = 6-32). (**b**) Crystalline cellulose quantification in elongation root EZ in control and streptol conditions. Data represent mean values, standard errors are indicated.

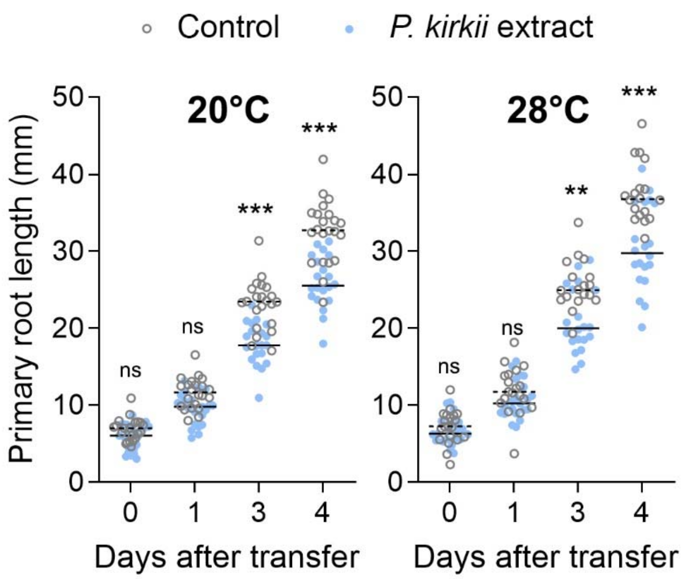

Supplementary Figure 10: The streptol phenotype is not enhanced by higher temperatures

Primary root growth of control-grown *Arabidopsis* seedlings transferred to fresh 1/2MS 1% sucrose plates and further grown at the indicated temperatures. Significant differences between treatments and water control were analysed in a two-sided unpaired t-test (** p-value < 0.01; *** p-value < 0.001; ns, not significant). Values represent the mean values of two biological replicates (10 plants per replicate); circles indicate data distribution, standard errors are indicated.

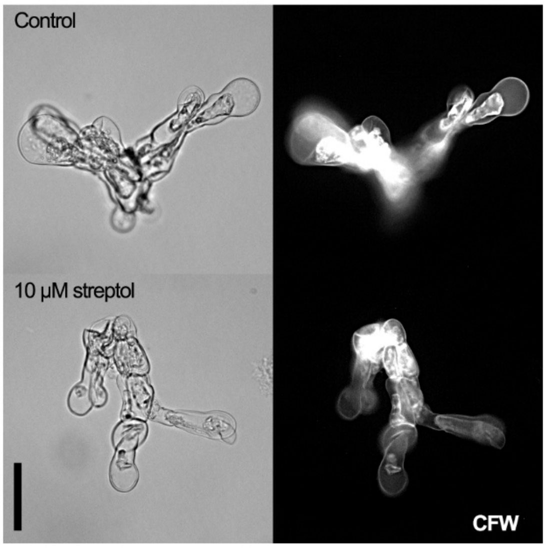

Supplementary Figure 11: Streptol does not affect planktonic *Arabidopsis* root cell cultures

Representative bright field and Calcofluor White (CFW) staining micrographs of control and streptol-treated *Arabidopsis* root cell suspension cultures. No significant differences were observed between treatment and control. Bar = 50 µm.

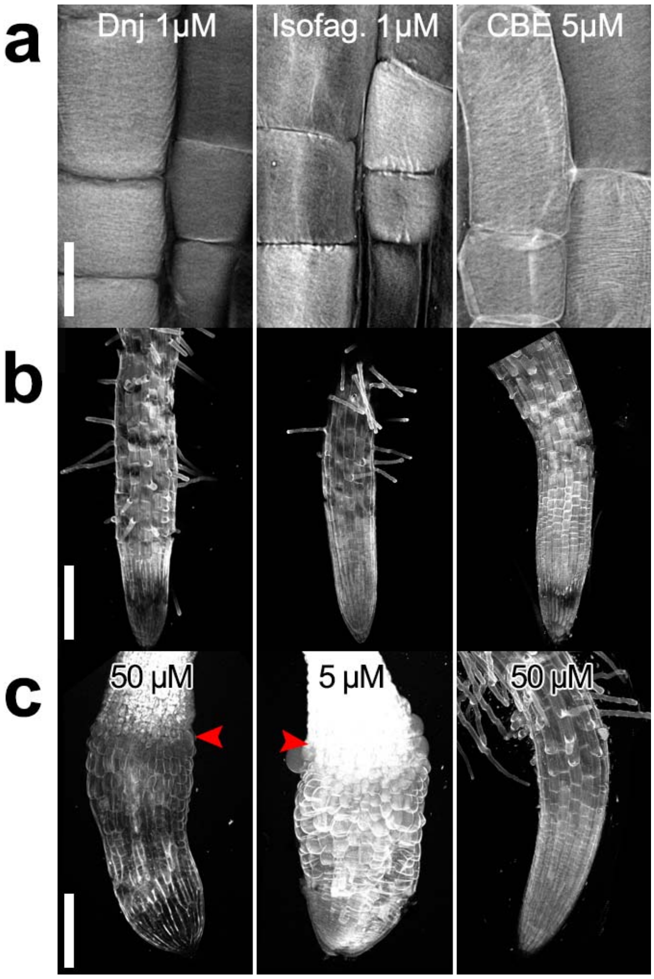

Supplementary Figure 12: Effect of GH inhibitors on *Arabidopsis* root cell walls

(**a**) Outer epidermal cell wall patterning in the elongation zone of *Arabidopsis* roots at the given growth-permissive inhibitor concentrations. Bar = 20 µm. (**b**) Primary root apex phenotypes corresponding to (a). Bar = 100 µm. (**c**) Root phenotype at root growth-restrictive concentrations. Arrowheads indicate the root-shoot junction. Bar = 100 µm. Cellulose microfibrils were stained with Direct Red 23. Representative maximum projections CLSM optical sections are shown. CBE, conduritol-β-epoxide.

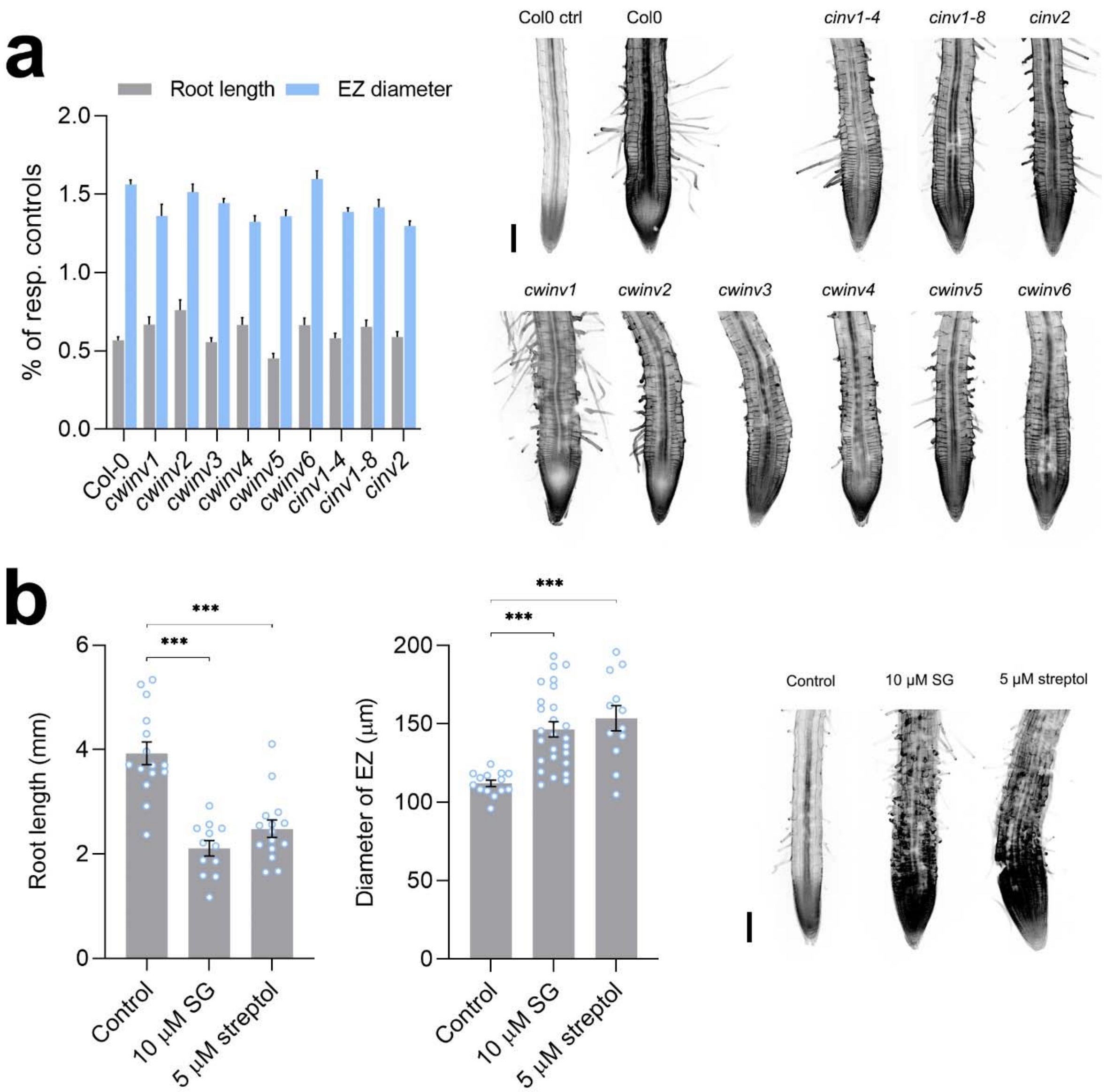

Supplementary Figure 13: Streptol glucoside does not target sucrose synthase or invertase activities

(**a**) Left, root length and root EZ diameter of invertase mutants grown in 10 µM SG conditions relative to respective untreated controls. Data represent mean values, standard errors are indicated. Right, corresponding primary root apex phenotypes. Bar = 100 µm. (**b**) Left, root length and root EZ diameter of the *sus1* mutant grown in streptol and SG conditions. Significant differences between treatments and water control were analysed in a one-way ANOVA with Dunnett's post-hoc test (*** p-value < 0.001). Values represent mean values; circles indicate data distribution, standard errors are indicated. Right, corresponding primary root apex phenotypes. Bar = 100 µm. Cellulose microfibrils were stained with Direct Red 23. Representative maximum projections of CLSM optical sections are shown.

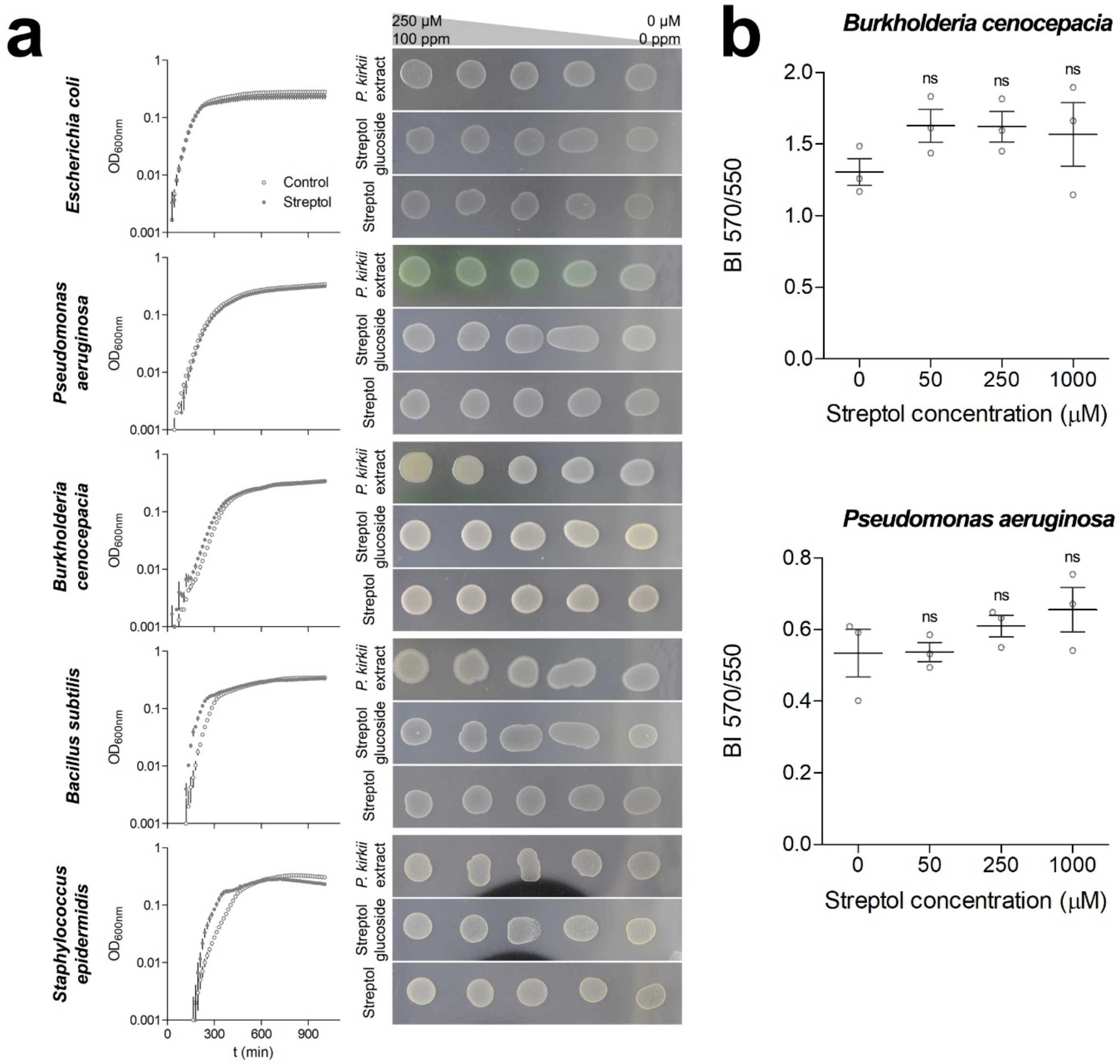

Supplementary Figure 14: Bacterial growth, EPS and biofilm production in streptol conditions

(**a**) Left, growth curves of selected bacterial species in liquid LB medium supplemented with 250 µM streptol. Data represent the mean of 3 biological replicates, standard errors are indicated. Similar results were obtained with streptol glucoside, (–)-streptol, (–)-streptol-β-glucoside and (–)-streptol-α-glucoside. Right, corresponding morphologies of bacterial colony grown on agar gradient yeast extract medium supplemented by the given concentrations of inhibitors. (**b**) Biofilm indices of selected bacterial species in streptol conditions. Significant differences between treatments and water control were analysed in a one-way ANOVA with Dunnett's post-hoc test (ns, not significant). Data represent mean values; circles indicate data distribution, standard errors are indicated. Similar results were obtained with streptol glucoside and (–)-streptol-β-glucoside.

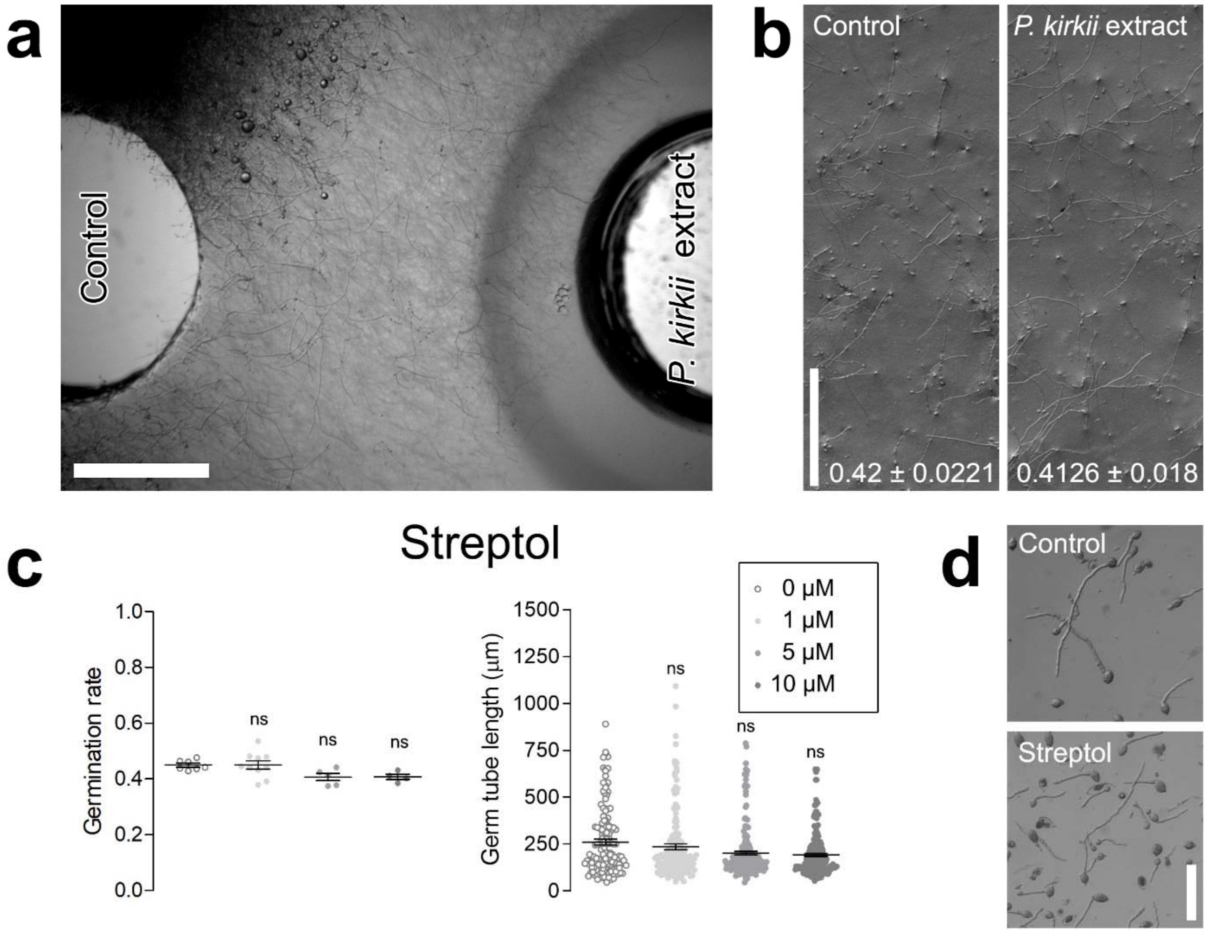

Supplementary Figure 15: Effect of streptol on the oomycete *Phytophthora infestans*

(**a**) Mycelial development in the presence of *P. kirkii* extracts; representative image. Agar wells were filled with undiluted extract or water control prior to the inoculation of a fresh *P. infestans* agar plug. The diffusion radius of the extract is visible. Bar = 5 mm. (**b**) Germination of *P. infestans* sporangia in 15 ppm *P. kirkii* extracts. Representative micrographs. The mean values of the germination rates of three biological replicates and standard errors are indicated. No significant differences between treatment and untreated control were found in a two-sided unpaired t-test. Bar = 1 mm (**c**) Sporangia germination rates and germ tube length in increasing concentrations of streptol. Significant differences between treatments and water control were analysed in a one-way ANOVA with Dunnett's post-hoc test (ns, not significant). Data represent mean values; circles indicate data distribution, standard errors are indicated. (**d**) Representative micrographs of germinated sporangia. Note the absence of abnormalities of germ tubes. Bar = 200 µm.

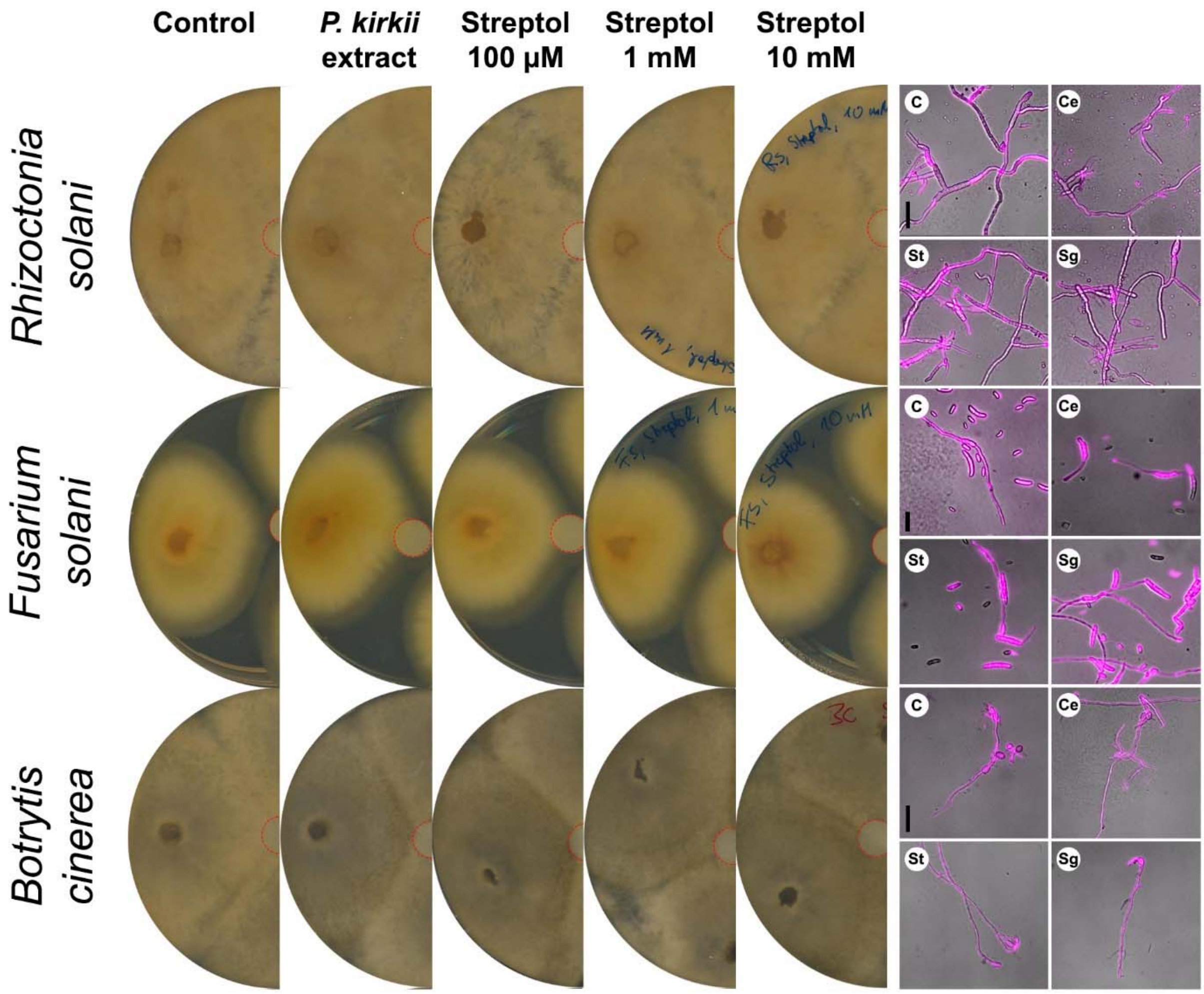

Supplementary Figure 16: Effect of streptol on fungal growth

Left, mycelial development in the presence of inhibitors; representative images. Red circles indicate filter discs soaked with the given inhibitor concentrations placed in the centre of the Petri dishes prior to the inoculation of a fresh mycelial agar plug. Right, spore germination or hyphal cuttings regeneration in the presence of inhibitors, representative micrographs. C, water control; Ce, undiluted *P. kirkii* crude extract; St, (+)-streptol; Sg, streptol glucoside. Calcofluor White fungal cell wall staining is shown in magenta. Bar = 20 µm.

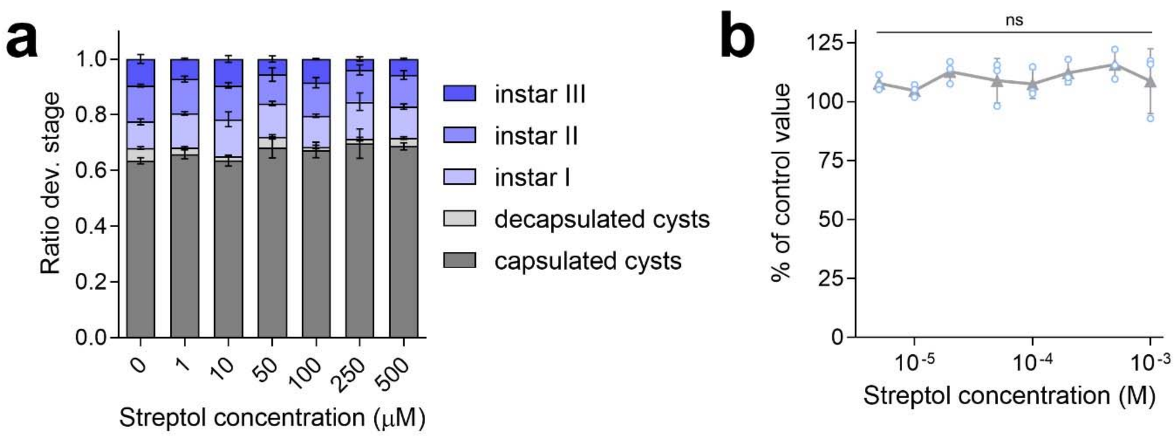

Supplementary Figure 17: impact of streptol on animal cells

(**a**) Proportions of *Artemia salina* developmental stages in increasing concentrations of (+)-streptol. Data represent mean values of three biological replicates (six for water controls) and 60 to 170 individuals were assessed in each treatment. Standard errors are indicated. No developmental abnormalities or significant differences in swimming motility of the larvae between treatments were observed. (**b**) human carcinoma LS 174T cells viability upon (+)-streptol treatment. Data represent mean values, circles indicate data distribution; standard deviations are indicated. Significant differences between treatments and water control were analysed in a one-way ANOVA with Dunnett's post-hoc test (ns, not significant). Microscopic observations of cells were performed after 2, 3 and 5 days of incubation; no cytotoxicity was detected at the given concentrations.

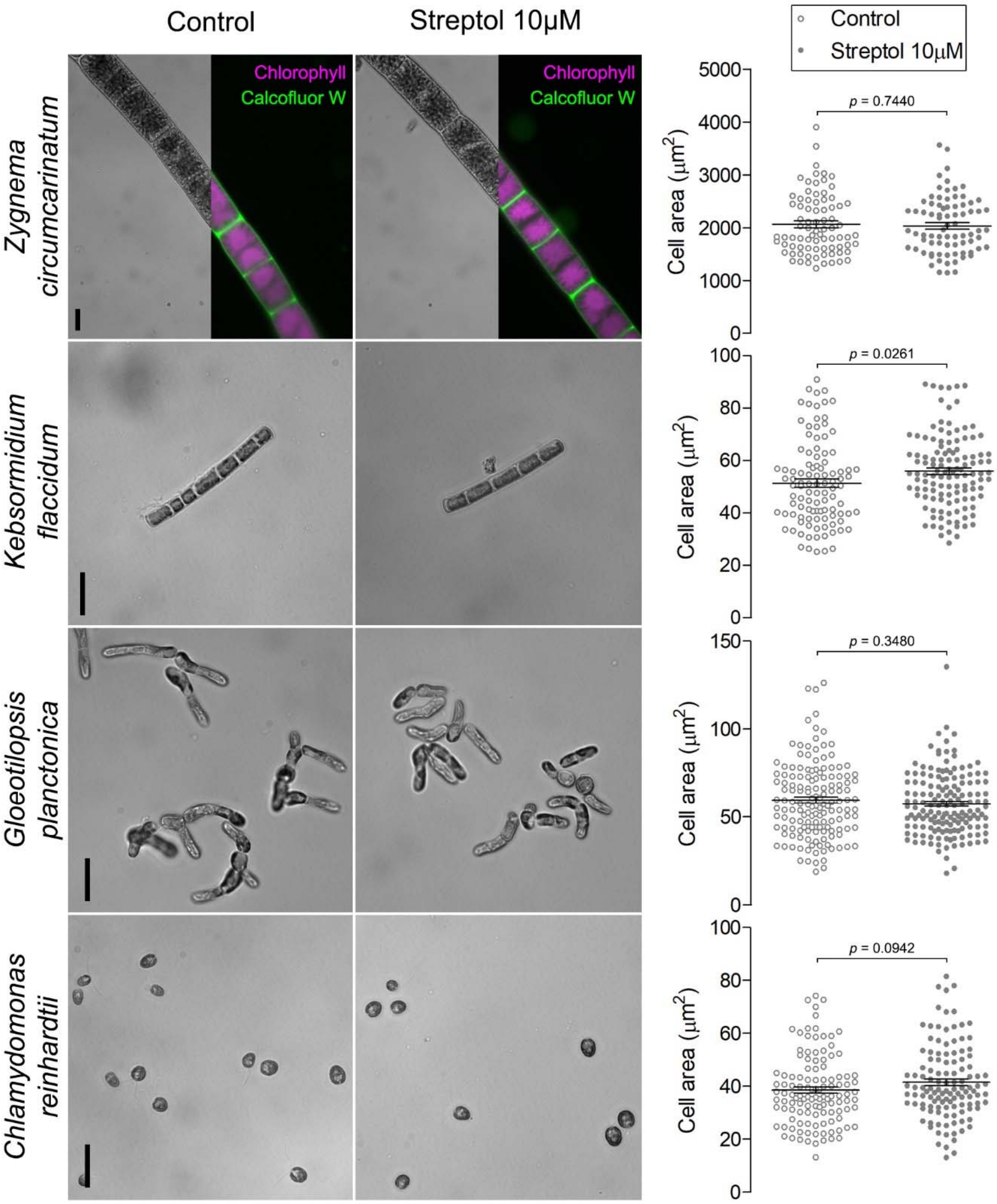

Supplementary Figure 18: Effect of streptol on algal growth

Left, representative micrographs of seven days algal growth in the presence of 10 µM streptol. Fluorescence channels are shown for *Zygnema* samples to appreciate chlorophyll content and cell wall staining. Bar = 20 µm. Right, cell area measured from micrographs for each treatment. Significant differences between treatment and water control were analysed in a two-sided unpaired t-test (*p*-values are indicated). Data represent the mean values of 3 biological replicates; circles indicate data distribution, standard errors are indicated. No notable difference in *C. reinhardtii* swimming motility was observed.

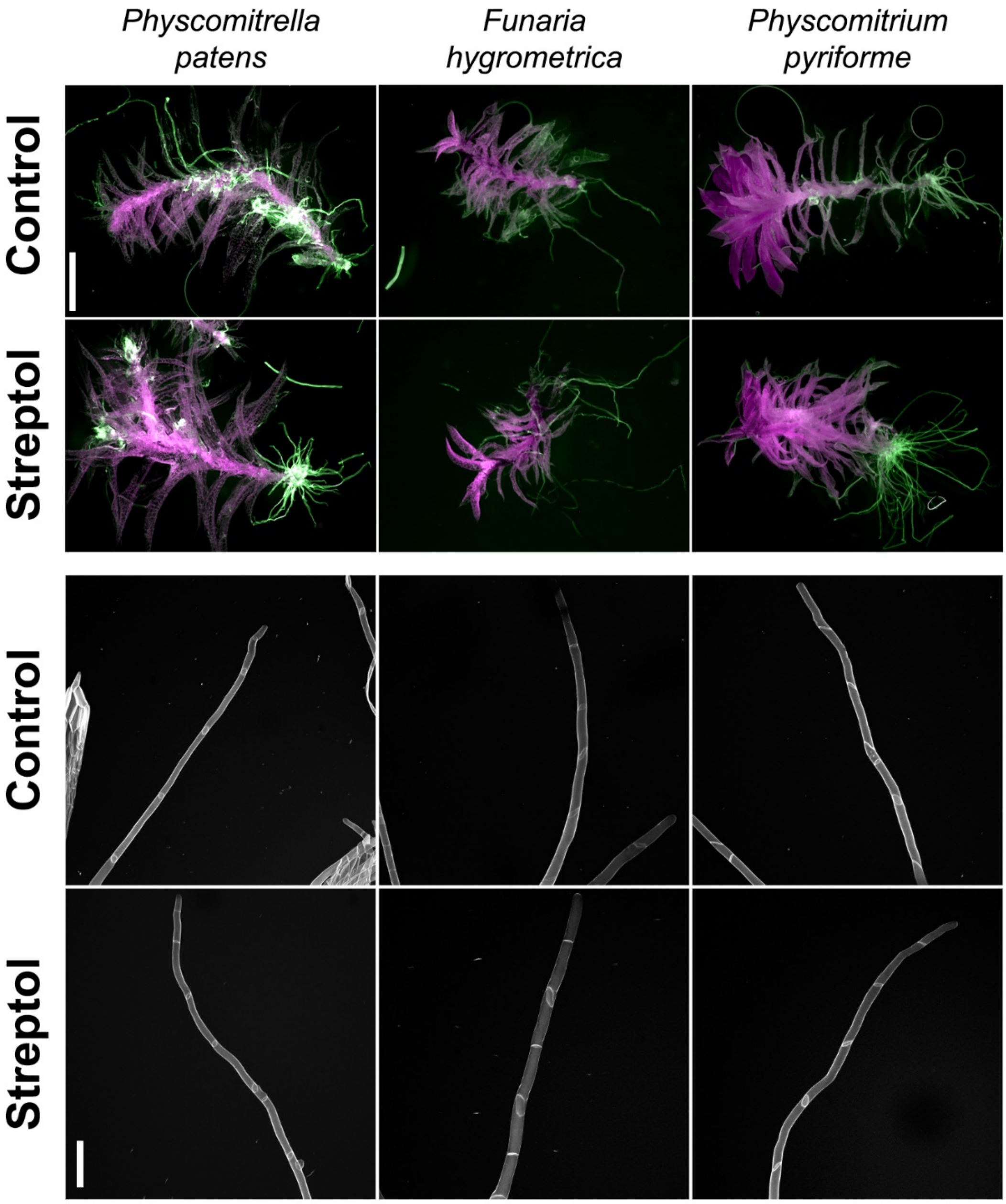

Supplementary Figure 19: Effect of streptol on mosses

Up, one month-old gametophores grown on solid medium in the presence of 10 µM streptol; representative micrographs. Chlorophyll autofluorescence is shown in magenta, Calcofluor White cell wall staining is shown in green. Bar = 1 mm. Bottom, morphologies of corresponding rhizoids, Calcofluor White cell wall staining. Representative micrographs. Bar = 100 µm.

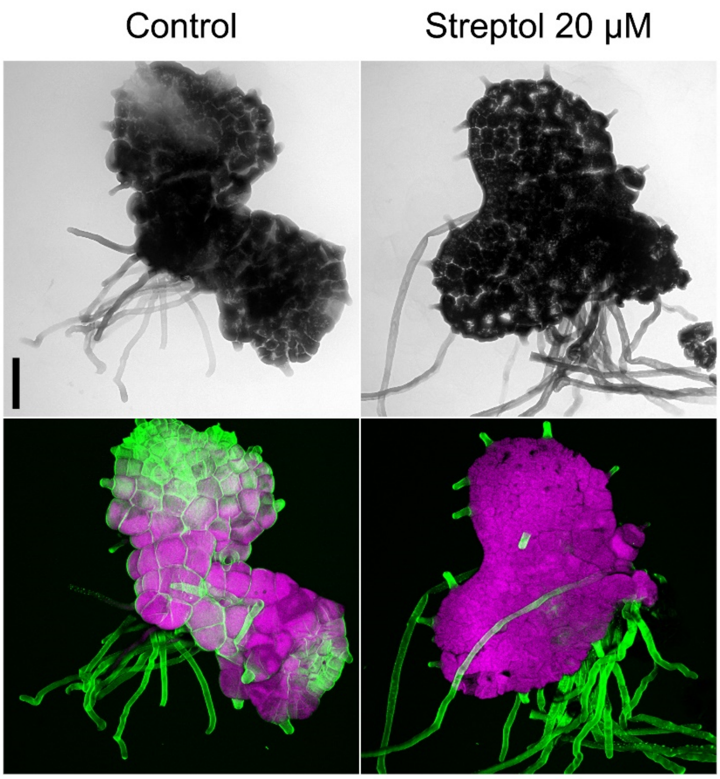

Supplementary Figure 20: Effect of streptol on the fern *Ctenitis submarginalis*

One month-old cordate gametophytes grown on solid medium in the presence or absence of 20 µM streptol; representative micrographs. Chlorophyll autofluorescence is shown in magenta, Calcofluor White cell wall staining is shown in green. Bar = 100 µm. No notable differences in gametophyte development or rhizoid morphology were observed between treatment and water control.

Supplementary Table 1. List and origin of plant species used in this study.

Plant species	Source
Charophytes	
Zygnema circumcarinatum 698-1a	The Culture Collection of Algae at Goettingen University
Klebsormidium flaccidum 2307	The Culture Collection of Algae at Goettingen University
Chlorophytes	
Gloeotilopsis planctonica 29.93	The Culture Collection of Algae at Goettingen University
Chlamydomonas reinhardtii 11-32b	The Culture Collection of Algae at Goettingen University
Bryophytes	
Physcomitrella patens 40001	International Moss Stock Center (IMSC)
Funaria hygrometrica 40017	International Moss Stock Center (IMSC)
Physcomitrium pyriforme 40059	International Moss Stock Center (IMSC)
Pteridophytes	
Ctenitis submarginalis	University of Zurich's Botanical Garden
Gymnosperms	
Sequoia sempervirens	Order from: http://www.sunshine-seeds.de/
Metasequoia glyptostroboides	Order from: http://www.sunshine-seeds.de/
Cupressus sempervirens	University of Zurich's Botanical Garden
Picea abies	University of Zurich's Botanical Garden
Angiosperms	
Poales	
Hordeum murinum	University of Zurich's Botanical Garden
Lagurus ovatus	University of Zurich's Botanical Garden
Aspargales	
Allium schoenoprasum	University of Zurich's Botanical Garden
Magnoliids	
Magnolia grandiflora	Order from: http://www.sunshine-seeds.de/
Caryophyllales	
Beta vulgaris	Local supermarket in Switzerland
Silene regia	Order from: http://www.sunshine-seeds.de/
Fabales	
Medicago sativa	Local supermarket in Switzerland
Brassicales	
Arabidopsis thaliana	University of Zurich's Botanical Garden
Raphanus sativus	Local supermarket in Switzerland
Brassica nigra	Local supermarket in Switzerland
Ericales	
Ardisia crenata	University of Zurich's Botanical Garden

Ardisia polycephala	University of Bonn’s Botanical Garden
Apiales	
Daucus carota	Local supermarket in Switzerland
Asterales	
Helianthus annuus	Local supermarket in Switzerland
Lactuca sativa var. *acephala*	Local supermarket in Switzerland
Solanales	
Solanum lycopersicum var. *matina*	Local supermarket in Switzerland
Lamiales	
Plectranthus scutellarioides	Order from: http://www.sunshine-seeds.de/
Gentianales	
Psychotria kirkii	University of Zurich's Botanical Garden
Psychotria capensis	Order from: http://www.sunshine-seeds.de/
Pavetta edentula	Order from: http://www.sunshine-seeds.de/
Hamelia macrantha	Order from: http://www.sunshine-seeds.de/
Kraussia floribunda	Order from: http://www.sunshine-seeds.de/
Gardenia cornuta	Order from: http://www.sunshine-seeds.de/

227

Supplementary Material

Streptol and streptol glucoside quantifications and standard curves. Dotted lines represent errors.

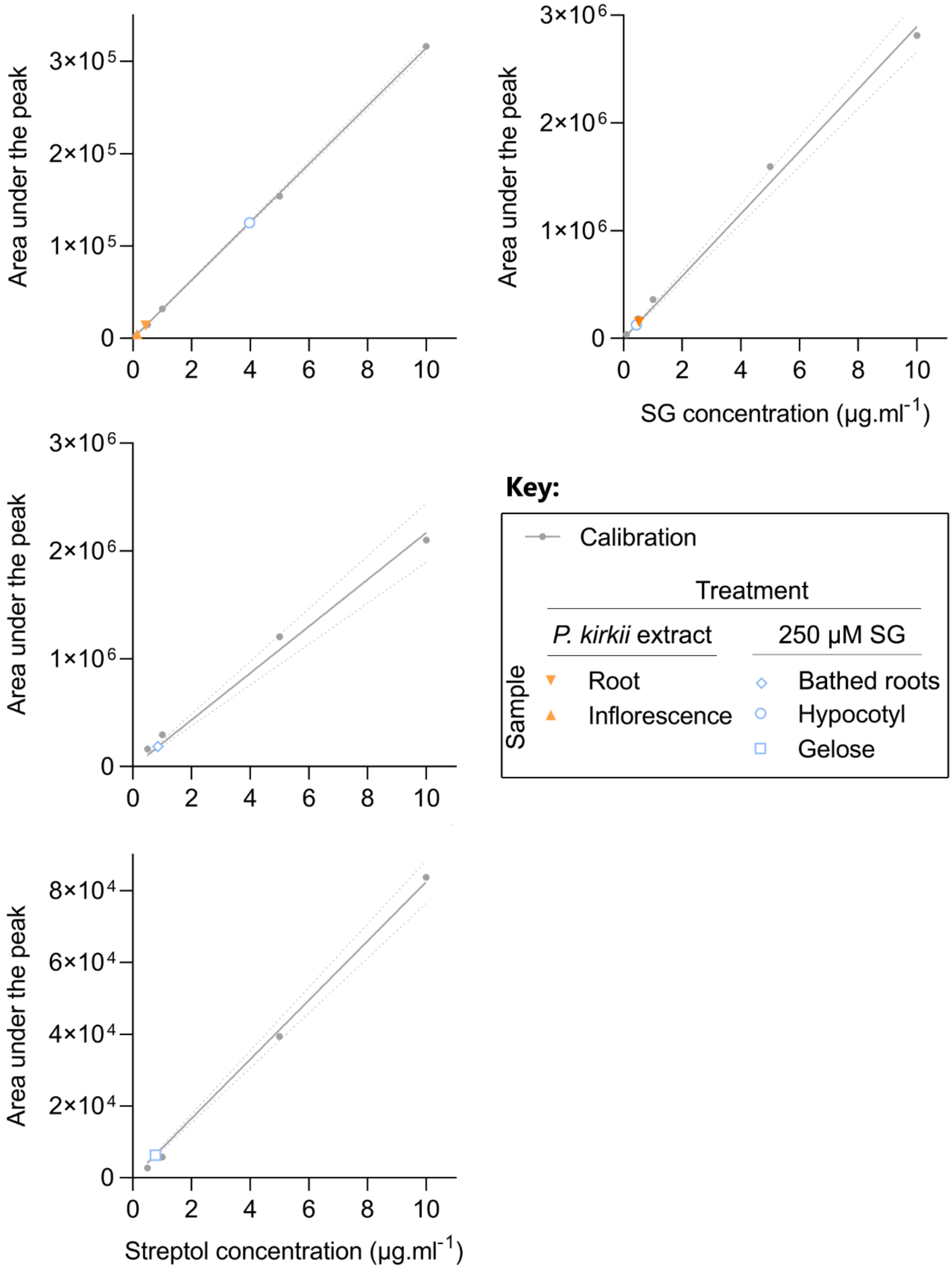

Arabidopsis lines used in this study:

Line	Identifier	References
prc1/cesa6	AT5G64740	[1]
cev1/cesa3	AT5G05170	[2]
rsw1/cesa1	AT4G32410	[3]
cwinv1	AT3G13790	[4,5]
cwinv2	AT3G52600	[5]
cwinv3	AT1G55120	[6]
cwinv4	AT2G36190	[7]
cwinv5	AT3G13784	homozygous SALK_201906C. D. Santelia pers. comm.
cwinv6	AT5G11920	[6]
cinv1	AT1G35580	[8]
cinv2	AT4G09510	[9]
stp1-1	AT1G11260	[10,11]
stp13-1	AT5G26340	[11,12]
stp1-1 stp13-1	-	[11]
stp1 stp4	AT3G19930	[11]
DR5::GFPm	-	[13]
pUBQ10::EYFP-TUB6	AT5G12250	[14]
pCYCB1;1::DB-GUS	-	[15]

Source publication:

1. Fagard, M. *et al.* PROCUSTE1 Encodes a Cellulose Synthase Required for Normal Cell Elongation Specifically in Roots and Dark-Grown Hypocotyls of Arabidopsis. *Plant Cell* **12**, 2409–2423 (2000).
2. Ellis, C. & Turner, J. G. The Arabidopsis mutant cev1 has constitutively active jasmonate and ethylene signal pathways and enhanced resistance to pathogens. *Plant Cell* **13**, 1025–1033 (2001).
3. Williamson, R. E. *et al.* Morphology of rsw1, a cellulose-deficient mutant of Arabidopsis thaliana. *Protoplasma* **215**, 116–127 (2001).
4. Von Schweinichen, C. & Büttner, M. Expression of a plant cell wall invertase in roots of Arabidopsis leads to early flowering and an increase in whole plant biomass. *Plant Biol.* **7**, 469–475 (2005).
5. Tymowska-Lalanne, Z. & Kreis, M. Expression of the Arabidopsis thaliana invertase gene family. *Planta* **207**, 259–265 (1998).
6. De Coninck, B. *et al.* Arabidopsis AtcwINV3 and 6 are not invertases but are fructan exohydrolases (FEHs) with different substrate specificities. *Plant, Cell Environ.* **28**, 432–443 (2005).
7. Ruhlmann, J. M., Kram, B. W. & Carter, C. J. Cell wall invertase 4 is required for nectar production in Arabidopsis. *J. Exp. Bot.* **61**, 395–404 (2010).
8. Lou, Y., Gou, J. Y. & Xue, H. W. PIP5K9, an Arabidopsis phosphatidylinositol monophosphate kinase, interacts with a cytosolic invertase to negatively regulate sugar-mediated root growth. *Plant Cell* **19**, 163–181 (2007).
9. Barratt, D. H. P. *et al.* Normal growth of Arabidopsis requires cytosolic invertase but not sucrose synthase. *Proc. Natl. Acad. Sci. U. S. A.* **106**, 13124–13129 (2009).
10. Sherson, S. M., Alford, H. L., Forbes, S. M., Wallace, G. & Smith, S. M. Roles of cell-wall invertases and monosaccharide transporters in the growth and development of Arabidopsis. *J. Exp. Bot.* **54**, 525–531 (2003).
11. Flütsch, S. *et al.* Glucose uptake to guard cells via STP transporters provides carbon sources for stomatal opening and plant growth . *EMBO Rep.* **21**, 1–13 (2020).
12. Nørholm, M. H. H., Nour-Eldin, H. H., Brodersen, P., Mundy, J. & Halkier, B. A. Expression of the Arabidopsis high-affinity hexose transporter STP13 correlates with programmed cell death. *FEBS Lett.* **580**, 2381–2387 (2006).
13. Ottenschläger, I. *et al.* Gravity-regulated differential auxin transport from columella to lateral root cap cells. *Proc. Natl. Acad. Sci. U. S. A.* **100**, 2987–2991 (2003).
14. Sugiyama, Y., Wakazaki, M., Toyooka, K., Fukuda, H. & Oda, Y. A Novel Plasma Membrane-Anchored Protein Regulates Xylem Cell-Wall Deposition through Microtubule-Dependent Lateral Inhibition of Rho GTPase Domains. *Curr. Biol.* **27**, 2522-2528.e4 (2017).
15. Colón-Carmona, A., You, R., Haimovitch-Gal, T. & Doerner, P. Spatio-temporal analysis of mitotic activity with a labile cyclin-GUS fusion protein. *Plant J.* **20**, 503–508 (1999).
